# Exploration of questionable backbone conformations in crystallographic structure models using a structural alphabet

**DOI:** 10.1107/S2059798325009301

**Published:** 2025-11-25

**Authors:** Clémence Sarrau, Marine Baillif, Lucas Mantel, Dounia Benyakhlaf, Shamima Peerbux, Leslie Regad

**Affiliations:** a Université Paris Cité, Unité de Biologie Fonctionnelle et Adaptative (BFA) CNRS UMR 8251 – In Silico Pharmacological Profiling (IsPP) INSERM U1133, F-75205Paris CEDEX 13, France; European Bioinformatics Institute, United Kingdom

**Keywords:** questionable conformations, crystallographic structures, structural alphabet, structural deformation

## Abstract

More than 80% of protein structure models in the Protein Data Bank have been solved using X-ray crystallography. Despite continuous improvements in this experimental technique, crystallographic structure models may still present artifacts related to the crystallization process as well as errors introduced during model building and refinement, even in high-resolution cases. Such limitations can alter atomic or residue positions, leading to local misconformations, local or domain rearrangements, and occasionally global distortions. In this study, we developed a protocol to locate residues with questionable conformations, where conformations may be uncertain, atypical or influenced by crystallographic modeling and refinement. To do so, we started from a set of 826 nonredundant X-ray protein structure models. Each X-ray model underwent an energy-minimization step that relaxes atomic geometry by reducing potential energy. Residues that exhibited different local conformations between the X-ray and minimized models were therefore considered as having questionable conformations. To identify them, we compared the X-ray and minimized models of each protein using the HMM-SA structural alphabet. Our results revealed that over 18% of the residues in the protein set have questionable conformations in their backbone. These conformations can occur either as isolated events within the protein sequence or can form patterns. Moreover, we observed that the frequency of questionable conformations per X-ray model was independent of factors such as the date of deposition, resolution or crystal system. Analysis of the properties of residues associated with questionable conformations revealed that they do not specifically occur in flexible or accessible regions. However, there is a correlation between questionable conformations and secondary structures, with a particular overrepresentation of residues with questionable conformations in α-helices. We then further investigated questionable conformations in the structure model of ligand-free HIV-2 protease (PR2). By combining our questionable conformation-detection protocol with molecular-dynamics simulation, we demonstrated that approximately half of the questionable conformations in PDB entry 1hsi correspond to local conformations that are sparsely sampled by PR2 during the molecular-dynamics simulation or are structural outliers detected by the wwPDB report. In addition, our results suggested that these questionable conformations may affect the position of the flaps, two β-sheets forming the top of the binding site. In PDB entry 1hsi, their relative arrangement appears atypical compared with MD simulations, raising questions about the biological relevance of this conformation. To conclude, we have developed a protocol to quantify and localize questionable backbone conformations in X-ray structure models, which can affect the interpretation of structural data.

## Abstract 2

We present a protocol to detect and characterize questionable backbone conformations in X-ray protein structure models. These conformations may arise from artifacts related to the crystallization process or from errors introduced during model building and refinement. Applied to 826 proteins and illustrated by a case study on HIV-2 protease, the analysis revealed that nearly one fifth of residues are affected and that these conformations may influence the fold of certain regions and raise questions about their biological relevance.

## 1. Introduction

The Protein Data Bank (PDB) contains over 217 000 protein structure models, with 183 000 resolved using X-ray crystallography (Berman *et al.*, 2000 ▸). These entries result from a complex process, from macromolecule purification to structure model deposition in the wwPDB, with each step introducing potential errors. Each step in the process introduces both systematic and random errors. These errors can result from experimental limitations or from modeling and refinement decisions. Several metrics quantify the quality of protein structure models, such as resolution, *R*_free_ (Brünger, 1992 ▸), clashscore (Chen *et al.*, 2010 ▸) and real-space *R*-value *Z*-score (RSRZ) outliers (Kleywegt *et al.*, 2004 ▸; Gore *et al.*, 2012 ▸), which are detailed in the wwPDB X-ray Validation Report. Each of these parameters plays a crucial role in assessing different aspects of the accuracy of a molecular model. The resolution parameter focuses on the ability to discern details in an X-ray crystallographic model. The *R*_free_ criterion evaluates the overall quality of the model by comparing experimental data with calculated data, particularly emphasizing the validation set to avoid overfitting. The clashscore quantifies steric clashes in the model, and the RSRZ score provides a local assessment of structural quality by evaluating how well individual residues fit the electron density. Automated validation tools assess structure model quality by applying geometric and stereochemical criteria to detect errors and inconsistencies. *MolProbity* (Chen *et al.*, 2010 ▸) is one of the most widely used tools for this task. It provides a detailed evaluation of model geometry, including interatomic distances, bond angles and torsion angles. It also generates electron-density maps and checks the placement of residues using the Ramachandran plot, offering suggestions for correcting geometric defects. *WHAT_CHECK* (Hooft *et al.*, 1996 ▸) is a comprehensive validation tool used to assess the stereochemical quality of structure models. It analyses geometric parameters such as bond lengths, bond angles, side-chain conformations and atomic clashes. The program also provides an overall quality score to identify potential errors or unusual features in the model. *PROCHECK* (Laskowski *et al.*, 1993 ▸), on the other hand, focuses on torsion-angle analysis and the Ramachandran plot, helping to identify residues in geo­metrically unfavorable conformations. *PDB-REDO* (Joosten *et al.*, 2009 ▸, 2014 ▸; Joosten & Vriend, 2007 ▸; van Beusekom *et al.*, 2018 ▸) is an automated platform designed to improve macromolecular crystallographic models through iterative re-refinement, rebuilding and validation. It optimizes stereochemistry, updates atomic displacement parameters and re-evaluates *R* factors and *R*_free_ values by reanalyzing electron-density maps. These automated tools are essential not only for validating initial models but also for refining existing structure models, ensuring their accuracy and reliability before they are used in biological and biomedical studies. Despite advancements in resolution techniques and in structure model validation, artifacts related to the crystallization process and errors introduced during model building and refinement persist in X-ray structure models, even at high resolutions. These issues affect the conformations of individual atoms or residues, and can extend to local or domain movements or even structure models with global misfolding (Davis *et al.*, 2008 ▸). Several factors can induce these structural deviations: limitations in the crystallographer’s subjective interpretation of experimental electron-density maps, data quality, experimental conditions, errors in the refinement process, crystallization artifacts, crystal packing and inherent molecular properties such as protein flexibility (Brändén & Jones, 1990 ▸; Jones & Kjeldgaard, 1997 ▸; Davis *et al.*, 2008 ▸). For example, PDB entries 1jsq, 1pf4, 1z2r, 1s7b and 2f2m correspond to models of the MsbA and EmrE transporters. These entries were moved to the PDB obsolete archive because their shapes roughly resembled the mirror image of the correct structure model (Tate, 2006 ▸; Dawson & Locher, 2006 ▸). Additionally, the chemical environment of the crystal (buffer salt concentration, pH *etc.*) can affect the conformation observed in the X-ray model. For example, high concentrations of lyotropic salts in the crystallization buffer of human protein kinase CK2 maintain the closed conformation of the kinase (Klopffleisch *et al.*, 2012 ▸; Srivastava *et al.*, 2018 ▸). Another example illustrating the impact of experimental conditions on protein conformation is the Bcl-xL protein. When resolved in the presence of detergents such as *n*-octyl-β-d-maltoside or exposed to pH 10, Bcl-xL undergoes structural rearrangements. In this state, the two α5 and α6 helices fuse into a single long helix (O’Neill *et al.*, 2006 ▸; Follis *et al.*, 2013 ▸; Tanaka *et al.*, 2013 ▸; Rajan, Choi, Baek *et al.*, 2015 ▸; Rajan, Choi, Nguyen *et al.*, 2015 ▸; Salam *et al.*, 2018 ▸). Crystal packing is another parameter that can introduce artifacts or deviations from biological conformations in X-ray models. It refers to the arrangement of individual biomolecules within a crystal lattice during crystallization for X-ray crystallography. As a result of crystallization, protein molecules in asymmetric units or neighboring symmetry-related molecules interact. These non­biological interactions, which are absent in functional molecules in cells, are referred to as crystal-packing artifacts (Tsuchiya *et al.*, 2008 ▸).

The impact of crystal packing on protein structures has been explored in various studies. One strategy consists of comparing X-ray structure models of the same protein resolved under different conditions (Heinz *et al.*, 1991 ▸; Zhang *et al.*, 1995 ▸; Bertrand *et al.*, 2000 ▸; Taylor *et al.*, 2001 ▸; Eyal *et al.*, 2005 ▸; Tsuchiya *et al.*, 2008 ▸; Regad *et al.*, 2017 ▸; Srivastava *et al.*, 2018 ▸; Triki *et al.*, 2019 ▸) or X-ray models independently crystallized by different groups (Martin *et al.*, 2008 ▸; Mei *et al.*, 2020 ▸). These studies revealed that packing effects can alter backbone or side-chain conformations, causing rigid-body motions of large structural units or loop conformational changes. For example, Eyal and coworkers compared 404 pairs of structure models of the same protein obtained from different crystals with identical forms (Eyal *et al.*, 2005 ▸). They showed that residues involved in crystal contacts are less mobile than other surface residues. They also found that crystal packing can modify water positions but does not affect ligand positions. To distinguish structural deformations induced by partner binding from those caused by experimental errors or crystallographic artifacts, Martin and coworkers used a set of 14 protein model pairs independently crystallized by different groups (Martin *et al.*, 2008 ▸). To more broadly explore the deformations caused by crystal packing, several studies compared X-ray and NMR models (Betts & Sternberg, 1999 ▸; Jacobson *et al.*, 2002 ▸; Eyal *et al.*, 2005 ▸; Garbuzynskiy *et al.*, 2005 ▸; Andrec *et al.*, 2007 ▸; Sikic *et al.*, 2010 ▸; Koehler Leman *et al.*, 2018 ▸; Mei *et al.*, 2020 ▸; Grigas *et al.*, 2022 ▸). For example, Mei and coworkers compared the cores of X-ray and NMR models for 21 proteins, revealing that NMR models are more tightly packed than the cores of X-ray models (Mei *et al.*, 2020 ▸). Unfortunately, only a few proteins in the PDB have been resolved by both NMR and X-ray crystallography. This makes it difficult to compare multiple NMR models with a single X-ray model. Other studies compared biological interfaces with nonbiological interfaces induced by crystal packing. They showed that packing contacts have smaller interfaces than biological contacts and that their amino-acid composition is indistinguishable from the rest of the protein surface. In addition, these contacts are less hydrophobic and contain fewer fully buried atoms (Janin & Rodier, 1995 ▸; Carugo & Argos, 1997 ▸; Carugo & Djinović-Carugo, 2012 ▸; Luo *et al.*, 2015 ▸). Several algorithms have been developed to predict whether an interface corresponds to a biological or crystallo­graphic interface (Krissinel & Henrick, 2005 ▸, 2007 ▸; Elez *et al.*, 2020 ▸; Liu *et al.*, 2014 ▸; Tsuchiya *et al.*, 2006 ▸, 2008 ▸; Zhu *et al.*, 2006 ▸; Bernauer *et al.*, 2008 ▸; Elez *et al.*, 2018 ▸). For example, the *PISA* method predicts the stability of an interface using a scoring function (Krissinel & Henrick, 2005 ▸, 2007 ▸). All of these studies have only explored artifacts in X-ray structure models arising from crystal packing without taking into account other types of deviations, or conformational changes located outside the interfaces. Additionally, they are often based on small data sets and focus on exploring either the global fold or side-chain deformations.

Detecting and understanding residues with questionable conformations in X-ray structure models is crucial when studying proteins. These conformations may reflect local uncertainties, modeling errors or artifacts arising from crystallographic conditions. In all cases, they can compromise structural interpretation and lead to misleading conclusions. In this study, we focused on locating and characterizing residues whose local conformations appear to be questionable in the backbone of X-ray structure models, using a large data set of 826 proteins. To identify such cases, we compared the local conformations of residues in X-ray models with those in the corresponding energy-minimized models. Energy minimization locally relaxes the atomic geometry by reducing steric clashes and strained conformations, without being constrained by crystallographic restraints. This procedure may therefore propose alternative conformations for residues affected by local uncertainties, actual inaccuracies (such as modeling errors and clashes) or deviations arising from crystallographic artifacts. Using the HMM-SA structural alphabet (Camproux *et al.*, 2004 ▸; Regad *et al.*, 2008 ▸), the X-ray and minimized models of proteins were encoded into sequences of structural letters, where each letter represents the fold of a four-residue fragment. Residues with questionable conformations, as used here, are defined as residues whose backbone adopts different local conformations in X-ray and minimized models, corresponding to different structural letters in the sequences derived from each model. Our results revealed that more than 18% of residues in the data set exhibit questionable backbone conformations. The proportion of such residues per X-ray model does not depend on protein length, crystal system or the date of model deposition. In contrast, we observed a moderate association with resolution and CATH classes. In particular, residues within helical regions were overrepresented among those with questionable conformations. Furthermore, we demonstrated that residues with questionable conformations are not preferentially located in flexible and solvent-exposed regions. The impact of the presence of questionable conformations on the global fold was explored using a case study of HIV-2 protease, an important target for treating HIV-2 infection. Our results showed a correspondence between residues with questionable conformations and those detected as structural outliers. Additionally, analysis of the HIV-2 protease structure during molecular-dynamics simulations revealed that residues with questionable conformations cause certain regions to adopt unsual conformations. Our study revealed that questionable conformations are common, particularly in structure models rich in α-helices. Our findings emphasize the need for caution when interpreting X-ray structure models and highlight the importance of thoroughly assessing their quality before use.

## 2. Materials and methods

### 2.1. Data collection

#### 2.1.1. The data set used to explore and quantify questionable conformations in X-ray models

We started with a set of 826 nonredundant X-ray protein structure models (less than 25% sequence identity) extracted from the PDB. This data set was named the Xray_826_ set. All models had a resolution better than 4 Å and more than 40 residues (Supplementary Fig. S1). They were in the free form, *i.e.* they were not complexed with a ligand or peptide and no metals were present in the structure models. Most structure models (75%) were monomers or dimers and contained fewer than 1200 residues. The X-ray model resolution distribution was centered around 2 Å and a large number of models (63%) were crystallized in the monoclinic or orthorhombic crystal systems (Supplementary Fig. S1). The distribution of CATH classes in the data set was similar to that of the CATH classification, with over 50% of structures classified as ‘alpha beta’ (Sillitoe *et al.*, 2021 ▸; Supplementary Fig. S1).

#### 2.1.2. The common data set between the Xray_826_ set and the PDB-REDO databank

The PDB-REDO databank (Joosten *et al.*, 2009 ▸, 2014 ▸; Joosten & Vriend, 2007 ▸; van Beusekom *et al.*, 2018 ▸; https://pdb-redo.eu) is a comprehensive and open-access repository that provides refined macromolecular structure models. These models, originating from the PDB, undergo an automated re-refinement process using the *PDB-REDO* procedure. This procedure enhances the quality and reliability of the original deposited models by applying advanced refinement tools and validating structural parameters. From an experimental model, the *PDB-REDO* procedure recalculates the atomic coordinates, optimizes stereochemical parameters and, when structure factors are available, improves the agreement between the model and the electron-density map. The platform also evaluates and adjusts *R* factors and *R*_free_ values, key indicators of model quality. In this study, we used these refined models extracted from the PDB-REDO databank as a reference to evaluate the ability of our protocol to identify residues with questionable conformations, particularly those associated with limitations in refinement or model interpretation. It is important to note, however, that the *PDB-REDO* re-refinement procedure does not affect the crystal-packing environment. As a result, conformational biases introduced by packing contacts remain unchanged.

We thus extracted the refined models of the Xray_826_ proteins from the PDB-REDO databank. Of these, 694 structure models were present in both the PDB and the PDB-REDO databank. This subset of 694 common models was denoted the Xray_694_ set. The 132 missing structures were most likely not available in the PDB-REDO databank because the corresponding experimental data (such as structure-factor files) may have been missing or incomplete in the original PDB entries. This lack of data would have prevented their re-refinement. For each protein of the Xray_694_ set, we have two models: the X-ray model extracted from the PDB (X-ray models) and the refined model extracted from the PDB-REDO databank (PDB-REDO models).

#### 2.1.3. HIV-2 protease structure model sets

**Figure 1:**
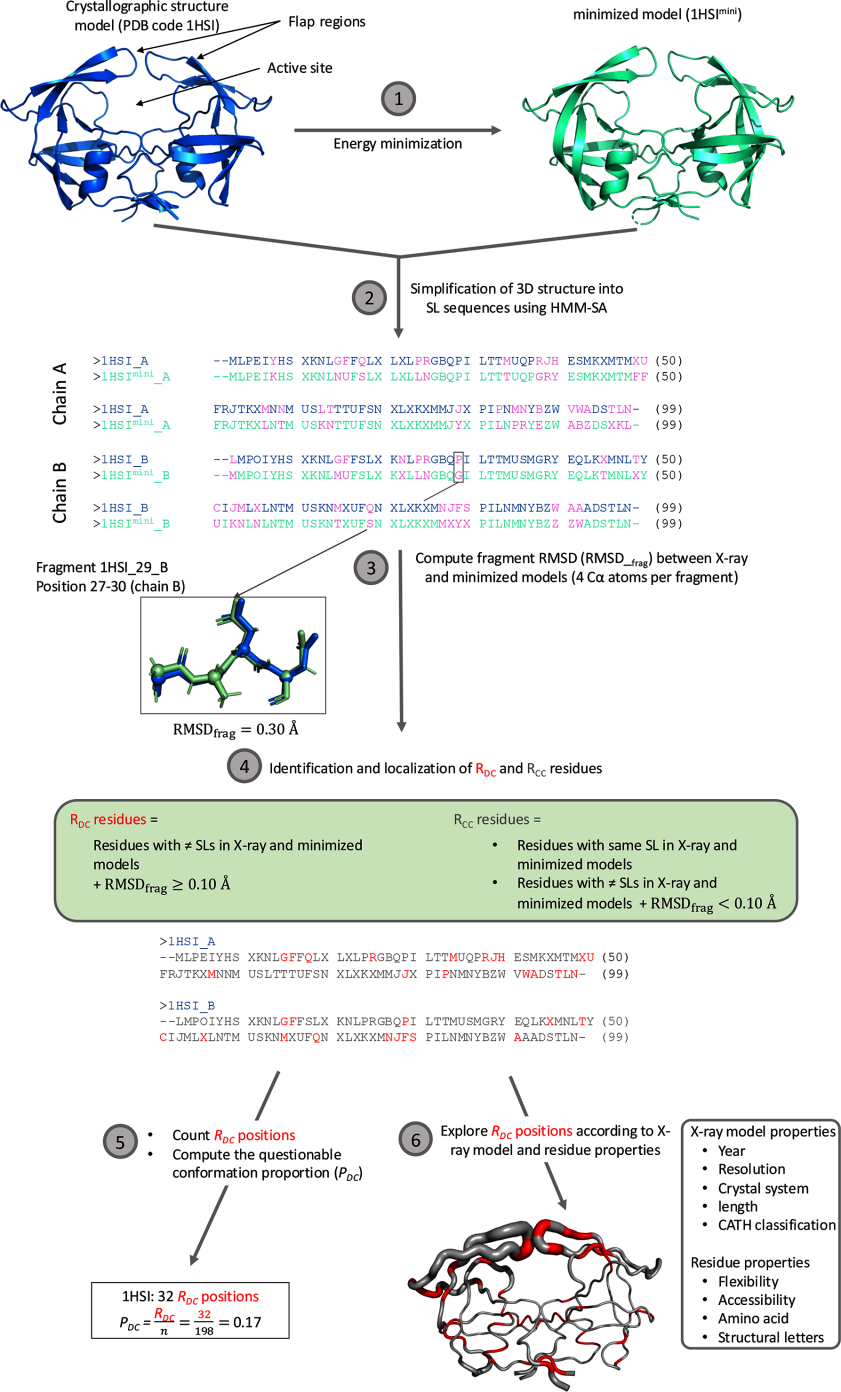
Protocol used to localize and analyze residues with questionable conformations in the PR2 structure model (PDB entry 1hsi). Step 1 corresponds to energy minimization of the X-ray model. Step 2 corresponds to simplification of the X-ray and minimized models into structural-letter sequences using HMM-SA (Camproux *et al.*, 2004 ▸). Each structural letter represents the geometry of a four-C^α^-atom fragment. Step 3 involves locating residues that have different structural letters in the sequences of X-ray and minimized models. These residues are highlighted in magenta in structural-letter sequences. In step 3, we compute the backbone-fragment r.m.s.d. (RMSD_frag_) between X-ray and minimized models, considering four-C^α^-atom fragments corresponding to residues with different letters in both models. Step 4: according to these results, residues with different structural letters between two models and RMSD_frag_ ≥ 0.10 Å are classified as *R*_DC_ residues. These *R*_DC_ residues are considered to be residues with questionable conformations. Other residues are classified as *R*_CC_ and considered to have well supported conformations. *R*_DC_ residues are highlighted in red in the structural-letter sequences of PDB entry 1hsi. In step 5, the number of *R*_DC_ residues per X-ray model is normalized by the length of the protein to compute the proportion of residues with questionable conformations per X-ray model, denoted as *P*_DC_. In step 6, we explore the relationship between *P*_DC_ and several X-ray model properties, including the year of deposition, the protein length, the number of chains, the resolution, the crystal system and the CATH class. We also examine the link between *R*_DC_ residues and residue flexibility, accessibility and the composition of amino acids and the structural letters. In step 6, PR2 is displayed as a putty cartoon, where the putty radius is proportional to the flexibility of residues, quantified by the *B*-factor values extracted from the PDB file. In all panels, *R*_DC_ residues are highlighted in red.

In the second step of this study, we explored in detail the questionable conformations in the structure model of HIV-2 protease (PR2) in its free form, *i.e.* not complexed with a ligand. PR2 is a crucial target for treating HIV-2 infection, as it represents one of the key enzymes essential for the replication of HIV-2. More precisely, PR2 is involved in the maturation of the virus by cleaving viral polyproteins into functional and structural components (Menéndez-Arias & Álvarez, 2014 ▸). This protein is a homodimeric aspartyl protease with 99 residues in each monomer. Its active site is situated at the interface of the two monomers (Fig. 1 ▸). It is covered by the two flap regions, which are β-sheets that close over the active site upon substrate binding. There are 19 structure models of PR2 available in the PDB, with 18 complexed with an inhibitor and one in a ligand-free form (PDB entry 1hsi; Chen *et al.*, 2014 ▸). In this study, we focused on this latter PR2 structure model without ligand.

To explore the conformational landscape of the ligand-free form of PR2, we generated a set of conformations using molecular-dynamics simulations with the *GROMACS* software (version 2022; Abraham *et al.*, 2015 ▸) and the Amber ff99SB-ILDN force field (Lindorff-Larsen *et al.*, 2010 ▸). Beginning with the X-ray model PDB entry 1hsi, we removed ions and water molecules. Next, we protonated the model at the O atom (OD1) of the aspartic acid at position 25 of chain *B* using the *PROPKA* software (Li *et al.*, 2005 ▸). The protein was then immersed in the center of a cubic water box (TIP3P) and placed at least 1.2 Å from the box edge. To neutralize the system, counterions were added. The neutralized system then underwent an energy-minimization step using the steepest-descent algorithm with 50 000 steps and a maximum force threshold of 1000.0 kJ mol^−1^ nm^−1^. Nonbonded interactions were truncated at a cutoff distance of 10 Å for the electrostatic twin-range cutoff and the van der Waals cutoff. The system was then equilibrated for 2 ns followed by a production step of 500 ns. These steps were performed at a pressure of 1 bar and a temperature of 300 K. The temperature and pressure were kept constant thanks to temperature coupling using the *V*-rescale method and pressure coupling controlled by the Parrinello–Rahman algorithm, respectively. The *LINCS* algorithm was used to constrain the covalent bonds of H atoms. Long-range electrostatic interactions were computed by the particle mesh Ewald (PME) method. The Verlet method was used for neighbor searching with a cutoff of 10 Å for both electrostatic and van der Waals interactions. Periodic boundary conditions were applied in three dimensions. A snapshot of the trajectory was saved every 1 ns, resulting in a set of 501 structure models referred to as MD models. The stability and the convergence of the system during the simulation are presented in Supplementary Fig. S2.

### 2.2. Location and quantification of residues with questionable conformations in X-ray structure models

To locate residues with questionable conformation in models of the Xray_826_ set, we used the two-step protocol developed in Ollitrault *et al.* (2018 ▸) and presented in Fig. 1 ▸.

#### 2.2.1. Step 1: energy-minimization step

The first step corresponded to an energy-minimization step for each model in the Xray_826_ set. The purpose of energy minimization was to optimize the conformation of the model by reducing structural strain or conflict and identifying a stable or quasi-stable state. This was achieved by decreasing the total potential energy of the system. Energy minimization generated a new structure model corresponding to a locally optimal state in terms of potential energy. Energy minimization modified residue conformations by reducing geometric anomalies in the protein structure model. These included steric clashes, excessively short interatomic distances or bond angles deviating from stereochemical expectations. The procedure also released constraints imposed by crystal packing, suggesting alternative conformations for residues influenced by lattice contacts. Residues that displayed different conformations between the X-ray and minimized models were considered to have questionable local conformations in the X-ray models.

Thus, in our protocol, we performed energy minimization of each Xray_826_ model with *GROMACS* (version 2022; Abraham *et al.*, 2015 ▸). During minimization, each system was solvated in a cubic box of explicit solvent (TIP3P water model) with a 10 Å buffer in each dimension. An appropriate number of chloride or sodium ions were added to produce a neutral charge in the system. Protein and water molecules were described using the AMBER99SB force field (Lindorff-Larsen *et al.*, 2010 ▸). Energy minimization was carried out using the steepest-descent algorithm with a maximum of 50 000 iterations. The PME method was adopted to treat the long-range electrostatic interactions (Darden *et al.*, 1993 ▸). The cutoff distances for the long-range electrostatic and van der Waals interactions were set to 10 Å. At the end of this step, a minimized model was generated for each X-ray model.

#### 2.2.2. Step 2: location and quantification of residues with questionable conformations in the Xray_826_ models

The second step was based on HMM-SA (Camproux *et al.*, 2004 ▸). HMM-SA is a library of 27 protein-backbone fragments of four C^α^ atoms, called structural letters (SLs) and labeled [A-Z,a]. It was obtained by classifying four-C^α^-atom fragments, overlapping by three residues, extracted from a non­redundant set of protein structures. The classification was performed using a hidden Markov model based on the geometry of the fragments and their succession in structures. HMM-SA has proved to be highly effective for describing and comparing protein structures (Regad *et al.*, 2008 ▸, 2017 ▸; Triki *et al.*, 2018 ▸, 2019 ▸; Ollitrault *et al.*, 2018 ▸). For example, we used HMM-SA to develop the *SA-conf* software (Regad *et al.*, 2017 ▸) dedicated to exploring and quantifying the structural variability of a protein target by comparing the local conformations of a set of its structure models. In particular, we employed *SA-conf* to investigate the structural deformation induced by inhibitor binding in HIV-2 protease (Triki *et al.*, 2018 ▸, 2019 ▸; Camproux *et al.*, 2025 ▸) and in the Bcl-xL target (Baillif *et al.*, 2025 ▸).

In this second step of our protocol, HMM-SA was used to simplify the X-ray and minimized models into two sequences of structural letters. During this simplification process, a structure model of *k* residues was simplified into a (*k* − 3) structural-letter sequence. Each structural letter corresponds to the local conformation of each four-C^α^-atom fragment (*i*, *i* + 1, *i* + 2, *i* + 3). The letter was assigned to the third residue (*i* + 2) of the fragment. At the end of this step, two structural-letter sequences were generated for each protein of the Xray_826_ set: one for the X-ray model and one for the minimized model. The two structural-letter sequences were then compared and allowed two types of residues to be distinguished. Firstly, residues having the same structural letter in both sequences. The energy-minimization step had no impact on the backbone conformation of these residues. Secondly, the residues that exhibited different structural letters between the X-ray and minimized models. Such residues corresponded to positions where the minimization step induced structural deformations, leading to distinct conformations in both models.

However, in HMM-SA, some structural letters are very close to each other, and a change from one to another may correspond to only a minor conformational adjustment. For example, on average, the backbone r.m.s.d. between a and other structural letters is only about 0.15 Å. To avoid overestimating the number of questionable residues, we further evaluated the difference between the X-ray and minimized fragments using the backbone r.m.s.d., named RMSD_frag_ (Fig. 1 ▸). Calculations were performed using the *MDAnalysis* package (Gowers *et al.*, 2016 ▸; Michaud-Agrawal *et al.*, 2011 ▸), considering only the C^α^ atoms of each fragment. Each fragment of the minimized model was superimposed onto its corresponding fragment in the X-ray model prior to RMSD_frag_ calculation. Only residues for which the RMSD_frag_ between the two four-C^α^-atom fragments exceeded 0.1 Å were retained. These residues were defined as *R*_DC_ residues, *i.e.* exhibiting questionable backbone conformations in the X-ray models. In contrast, those with an RMSD_frag_ below this threshold were considered unchanged and reclassified as *R*_CC_, similarly to residues that display identical structural letters in both models..

At the end of the process, the number of *R*_DC_ residues was determined per X-ray model, allowing a quantification of questionable conformations per X-ray model. This value was then normalized by the number of residues in the structure model to determine the questionable conformation proportion (*P*_DC_; Fig. 1 ▸).

#### 2.2.3. Identification of *R*_DC_ residues in the Xray_694_ data set

For the proteins in the Xray_694_ data set, we have two structure models: that extracted from the PDB (X-ray model) and that extracted from the PDB-REDO databank (PDB-REDO model). To identify residues with questionable backbone conformations in the data set of 694 structure models, we applied the same protocol as used for the X-ray/minimized model comparison. This procedure compared the X-ray model of each protein (retrieved from the PDB) with the corresponding refined model from the PDB-REDO databank using HMM-SA and RMSD_frag_. This comparison allowed the location of *R*_DC_ and *R*_CC_ residues in X-ray models of the Xray_694_ set. Then, the proportion of *R*_DC_ residues was determined for each Xray_694_ model. These results were then compared with those obtained from the X-ray/minimized model analysis. This comparison allowed us to examine whether the same residues tend to be identified as questionable in both approaches. The PDB/PDB-REDO comparison was nevertheless expected to yield fewer such residues, since crystal-packing effects are still present in the PDB-REDO models.

### 2.3. Determination of flexibility and accessibility of residues

#### 2.3.1. Characterization of residue flexibility

Each residue was categorized as either rigid or flexible based on its *B*-factor value, following a methodology similar to those of Karplus & Schulz (1985 ▸) and Triki *et al.* (2018 ▸, 2019 ▸). The *B*-factor value is indicative of the degree of isotropic smearing of electron density around the center of the residue (Parthasarathy & Murthy, 1997 ▸). Initially, for a protein, the *B*-factor for each atom was extracted from its corresponding PDB file representing the X-ray model. Subsequently, the average *B*-factor for each residue was computed based on all atoms and normalized using the overall average *B*-factor and standard deviation of the protein. Flexible residues were defined as those with a normalized *B*-factor greater than 0, while rigid residues were those with a normalized *B*-factor smaller than 0 (Karplus & Schulz, 1985 ▸; Triki *et al.*, 2018 ▸, 2019 ▸).

#### 2.3.2. Characterization of the accessible surface area (ASA)

We differentiated accessible and buried residues in each structure model according to their ASA value. To achieve this, the relative ASA (rASA) value was computed for each residue in each X-ray model. The calculations were performed for all atoms using the *NACCESS* software (Hubbard & Thornton, 1993 ▸) with a radius probe sphere of 1.4 Å. The higher the ASA value of a residue, the more accessible it is. Residues with a rASA value higher than 20% were defined as accessible, while others corresponded to buried residues (Eyal *et al.*, 2005 ▸).

#### 2.3.3. Location of structural asymmetric residues

We defined residues exhibiting structural asymmetry as those adopting different local conformations in two chains with the same amino-acid sequence within one structure model. To identify such residues in the 309 homo-oligomers from the Xray_826_ set, we employed the protocol developed in Triki *et al.* (2018 ▸). This method relies on HMM-SA to extract and compare local conformations between chains. HMM-SA is used to simplify both chains into sequences of structural letters. The structural letters of the two chains are then compared at each position. Residues that exhibit the same structural letter, *i.e.* the same local conformation, in both chains are classified as symmetric, while those with different structural letters, and therefore different local conformations, are considered structurally asymmetric. This protocol was applied to locate structurally asymmetric residues in the 309 homo-oligomers from the Xray_826_ set by comparing the local conformations of 1570 pairs of chains. As a result, we identified 60 719 structurally asymmetric residues and 161 568 structurally symmetric residues according to the HMM-SA-based definition. These numbers should be considered an estimation, as within the error margin of crystallographic structure models a change in structural letter is not necessarily significant.

### 2.4. Extraction of protein pockets and protein–protein interfaces

#### 2.4.1. Identification of pocket residues

Ligand-binding sites of each Xray_826_ structure model were estimated by detecting residues involved in surface pockets using the geometry-based software *fpocket* (Le Guilloux *et al.*, 2009 ▸). This software examines all of the protein cavities without information about ligands by decomposing the 3D protein structure model into Voronoi polyhedra. In the Xray_826_ set, a model contained, on average, 29 ± 42 pockets, with a maximum of 763 pockets in a structure model with 20 chains. This large variability reflects the structural diversity of the Xray_826_ data set, which includes proteins ranging from small monomeric enzymes to large multichain assemblies. It also arises from the sensitivity of *fpocket*, which detects not only major binding cavities but also small and shallow surface pockets (Le Guilloux *et al.*, 2009 ▸; Schmidtke *et al.*, 2010 ▸). Based on these pocket sets, we defined a residue involved in pockets, named a pocket residue, as a residue that had at least one atom in one pocket.

#### 2.4.2. Identification of residues involved in protein–protein interfaces

We extracted residues involved in protein–protein interfaces using the *PRODIGY-crystal* software (Jiménez-García *et al.*, 2019 ▸) from the 467 oligomers of the Xray_826_ set. *PRODIGY-crystal* enables the distinction between biological and crystal interfaces using a random forest predictor based on residue contacts and interaction energetic features (residue contacts per amino-acid type, contact density/interface). According to the results, we classified residues into three classes: (i) residues involved in a biological interface, (ii) residues involved in a crystal interface and (iii) residues located outside any interface. In the Xray_826_ set, 60% of oligomers contained at least one crystal interface. At the residue level, most of the oligomer residues (78 ± 16%) were outside any interface, whereas 15 ± 18% were in a biological interface and 7 ± 8% were in crystal interfaces.

### 2.5. Statistic approches

To compare the properties of *R*_DC_ and *R*_CC_ residues, we conducted statistical tests using the *R* software (*R* version 4.0.2; R Core Team, 2020 ▸) and interpreted them with a significance level α set at 5%. To enhance the interpretation of each test, the computation of *p*-values was complemented by calculating effect sizes. To compare the average values of flexibility and ASA between *R*_DC_ and *R*_CC_ residues, we performed Student’s *t*-tests (T-tests) and computed Cohen’s *d* parameter to quantify effect sizes (Cohen, 1988 ▸). Cohen’s *d* parameter indicates the standardized difference between the two means. Effect sizes were interpreted as very small (Cohen’s *d* < 0.2), small (0.2 ≤ Cohen’s *d* < 0.5), medium (0.5 ≤ Cohen’s *d* < 0.8) or large (Cohen’s *d* > 0.8). To explore the link between questionable conformations and CATH classes and crystal system, we compared the average value of *P*_DC_ accross CATH classes and crystal systems using Kruskal–Wallis tests. The effect sizes of Kruskal–Wallis tests were quantified by computing the η^2^ parameter, which indicates the variance in ranks attributable to group differences (Cohen, 1988 ▸). The interpretation of η^2^ values is as follows: η^2^ ≃ 0.01 indicates a small effect, η^2^ ≃ 0.06 indicates a medium effect and η^2^ ≃ 0.14 indicates a large effect. In the case of significant Kruskal–Wallis tests, we further analyzed the data by performing *post hoc* Dunn’s tests. The exploration of the link between *P*_DC_ value and the year of model entry, the protein length, the number of chains and the resolution of the crystallographic data was performed by computing the Pearson correlation coefficient. This parameter was also calculated to analyze the relationship between pattern and protein length or resolution of crystallographic data. Finally, χ^2^ tests were used to study the relationship between residue types (*R*_DC_ and *R*_CC_ residues) and pocket or interface location. These tests were accompanied by calculating Cramér’s *V* value, which quantifies the strength of association between two categorical variables (Cramér, 1946 ▸). A Cramér’s *V* value varies between 0 and 1; the closer Cramér’s *V* value is to 1, the stronger the relationship between the two categorical variables.

## 3. Results

### 3.1. Detection of residues with questionable conformations in the Xray_826_ model set

In this study, we investigated questionable conformations in a set of 826 X-ray structure models, which may arise from modeling inaccuracies, refinement limitations or crystal-packing effects. To identify and localize residues with questionable conformations in the Xray_826_ set, we first detected residues that exhibited different structural letters between the X-ray and minimized models. In total, 114 595 residues were identified in the Xray_826_ set, representing 31% of all residues. Surprisingly, three proteins displayed complete structural conservation between the X-ray and minimized models, with no residues showing different structural letters. These proteins contain between 85 and 1068 residues with resolutions ranging from 1.6 to 2.4 Å. One possible explanation for the absence of structural deviation is an issue during the minimization step.

**Figure 2:**
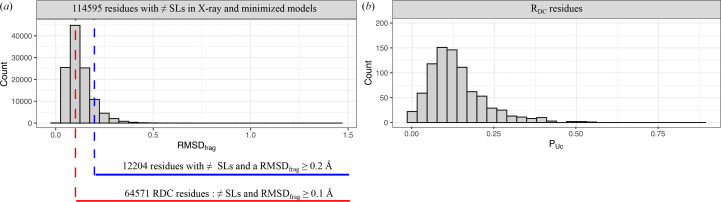
Identification of residues with questionable conformations based on HMM-SA and RMSD_frag_. (*a*) Distribution of RMSD_frag_ for all residues exhibiting different structural letters (SLs) across X-ray and minimized structure models. The vertical dashed red and blue lines indicate RMSD_frag_ thresholds of 0.1 and 0.2 Å, respectively. *R*_DC_ residues correspond to residues exhibiting different structural letters between X-ray and minimized models and an RMSD_frag_ higher than 0.1 Å. (*b*) Distribution of *P*_DC_ values within the Xray_826_ data set.

To further characterize residues with questionable conformations in the Xray_826_ set, we next calculated the RMSD_frag_ between the C^α^ atoms of the fragments corresponding to different structural letters between the X-ray and minimized structures (Fig. 2 ▸*a*). This step allowed us to quantify the local backbone deviations associated with structural-letter changes and to assess whether these differences reflect meaningful conformational variations or minor geometric deviations. The RMSD_frag_ values ranged from 0.012 to 1.457 Å, with a mean of 0.123 ± 0.065 Å. More than half of the selected residues (56%) had RMSD_frag_ ≥ 0.1 Å. These residues displayed measurable backbone deviations. Among these, 11% (corresponding to 3% of all residues) showed more pronounced backbone deformations, with RMSD_frag_ > 0.2 Å. We therefore defined residues with questionable conformations as those exhibiting different local conformations between the X-ray and minimized models, *i.e.* residues with different structural letters and an RMSD_frag_ > 0.1 Å. These residues, denoted *R*_DC_, represented 18% of all residues in the Xray_826_ set.

**Figure 3:**
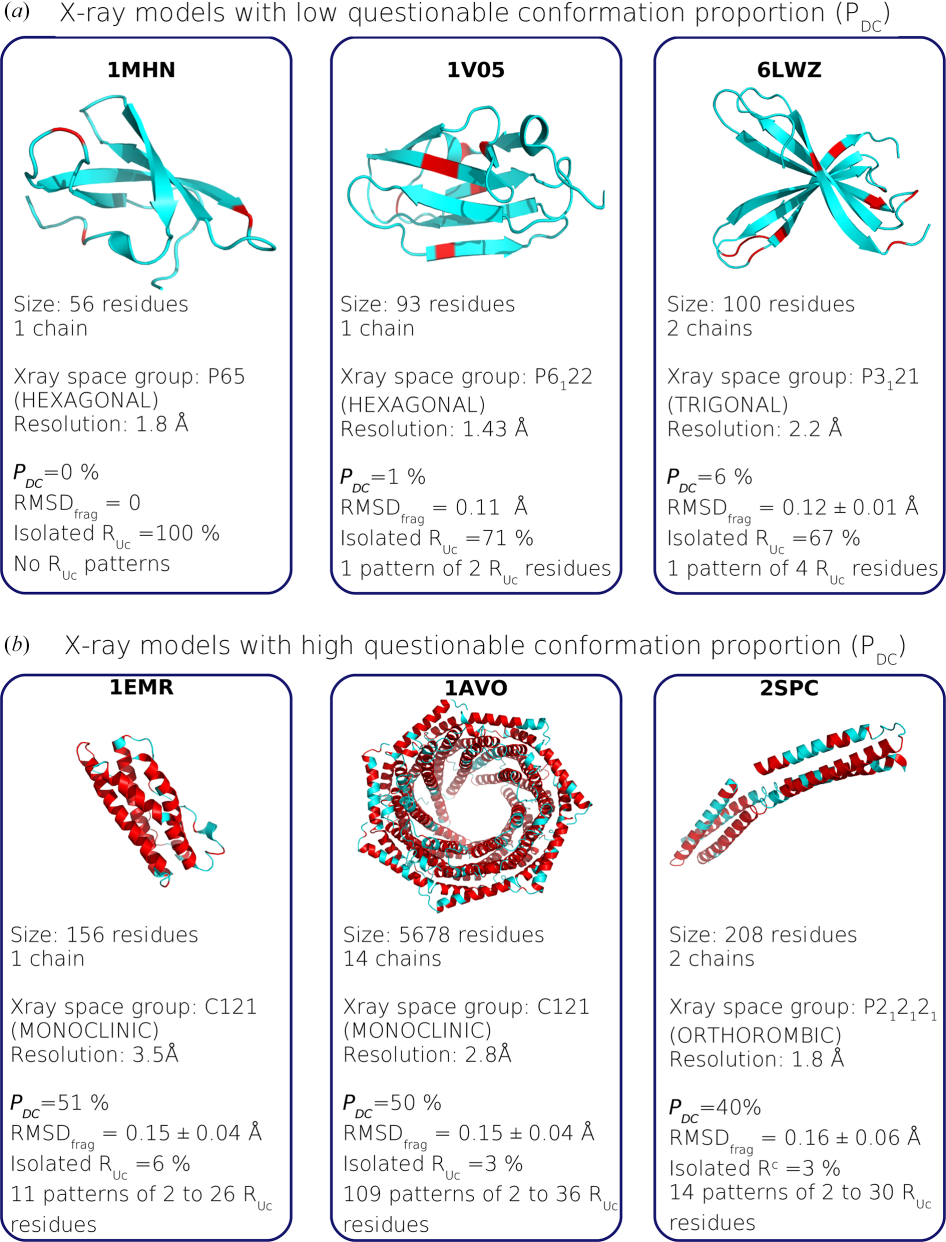
Illustration of X-ray models with high and weak *P*_DC_ values. For all examples, the protein is displayed in cartoon mode, where *R*_DC_ residues are highlighted in red. For each protein, the following information is indicated: the length, the number of chains, the crystallographic space group and the resolution. We also provide the *P*_DC_ value, the mean r.m.s.d. of the *R*_DC_ residues (average ± standard deviation), the proportion of isolated *R*_DC_ residues and the number of *R*_DC_ residues forming patterns and their length.

For each protein, we computed the questionable conformation proportion, notated *P*_DC_. In the Xray_826_ set, the *P*_DC_ values varied from 0 to 53%, with an average of 13.5% (±8.6%) (Fig. 2 ▸*b*). Most proteins (81%) exhibited a *P*_DC_ value below 20%. In contrast, five proteins displayed a *P*_DC_ value higher than 50%, meaning that at least half of their residues had questionable conformations. Fig. 3 ▸ illustrates X-ray models with low and high *P*_DC_ values. It shows that models with few questionable conformations were small with a lot of β-strands. In contrast, those with many questionable conformations were predominantly composed of α-helices.

### 3.2. Differences in structural-letter assignments for *R*_DC_ residues

**Figure 4:**
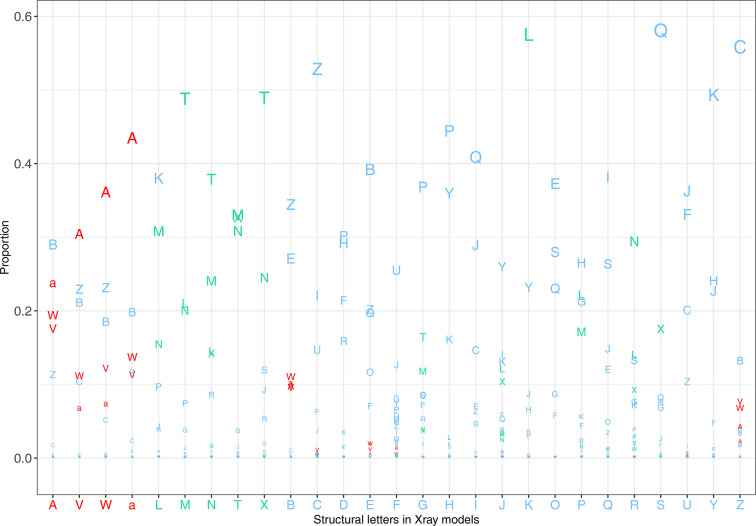
Proportion of each SL_Xray_–SL_minimized_ pair for all *R*_DC_ residues. An SL_Xray_–SL_minimized_ pair is composed of the structural letter in the X-ray model (SL_Xray_) and the structural letter in the corresponding minimized model (SL_minimized_). The *x* axis shows the 27 structural letters in X-ray models (SL_Xray_). The *y* axis indicates the proportion of occurrence of each SL_Xray_–SL_minimized_ pair in the Xray_826_ data set. Structural letters in the graphic correspond to the structural letters in minimized models (SL_minimized_). The height of the letters is proportional to the proportion of the SL_Xray_–SL_minimized_ pair. The greater the proportion of the SL_Xray_–SL_minimized_ pair (the larger the SL_minimized_ letter in the graph), the more the letter in the X-ray model (SL_Xray_) was deformed by the letter SL_minimized_ in the minimized models. Structural letters are colored according to the secondary structure: α-helix letters in red, β-strand letters in green and loop letters in blue.

To further investigate the nature of measurable backbone deviations, we analyzed the structural letters assigned to each *R*_DC_ residue in the X-ray and minimized models. For each residue, the structural letter in the X-ray model was compared with that in the corresponding minimized model, generating SL_Xray_–SL_minimized_ pairs. Fig. 4 ▸ provides the proportions of each SL_Xray_–SL_minimized_ pair. For example, more than 55% of the structural letters Z, K and S were deformed into the C, L and Q structural letters after minimization (Fig. 4 ▸). Overall, most structural letters were transformed into letters corresponding to the same or a closely related secondary structure (Fig. 4 ▸). For instance, over 56% of α-helix letters (A, a, V and W) were converted into other α-helix structural letters. In particular, 43% of a, 30% of V and 36% of W residues were transformed into the canonical α-helix letter A. Interestingly, the A letter itself was most frequently converted into the B letter (28%), which does not correspond to an α-helix but to a closely related conformation; nearly as often, it was converted into the a letter (23%). Similarly, the energy-minimization step induced deformation in β-strands without impacting the secondary structures. More than 77% of M, N, T and X letters were deformed into β-strand structural letters after minimization. For the L letter (a β-strand letter), 45% were changed into β-strand structural letters, while 37% became K, a letter closely related to β-strand structural letters. This analysis indicates that most structural changes observed between the X-ray and minimized models involve local conformations that remain close in structural space. This suggests that the questionable conformations in the X-ray models are not drastically altered by minimization.

### 3.3. Exploration of residues with questionable conformations in relation to the properties of X-ray structure models

**Figure 5:**
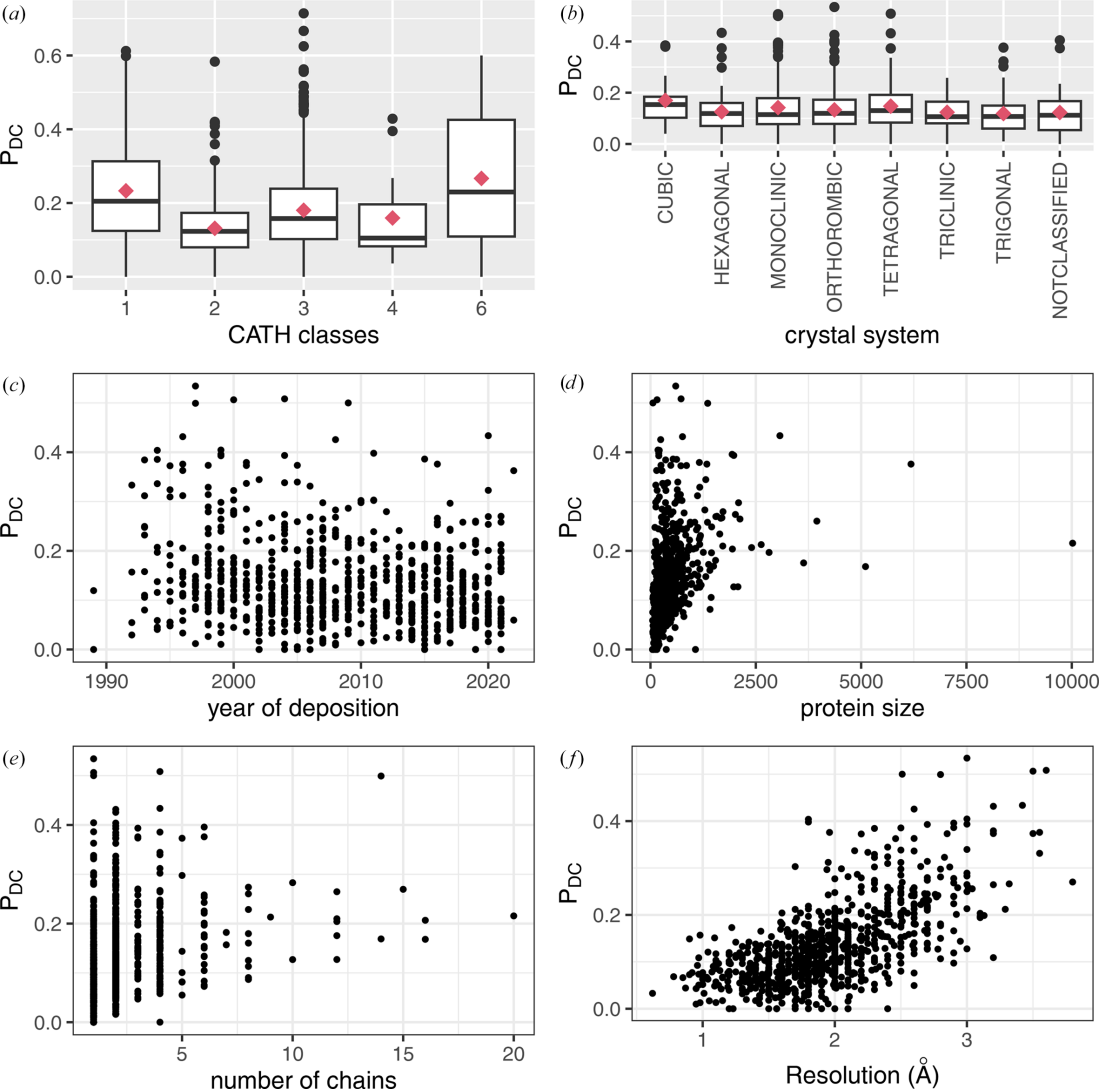
Exploration of the link between *P*_DC_ values and X-ray model properties: the CATH class (*a*), crystal system (*b*), year of deposition (*c*), length (*d*), number of chains (*e*) and resolution (*f*).

To better understand the occurrence of questionable conformations in X-ray models, we investigated the link between the *P*_DC_ value and several properties of X-ray structure models. Firstly, we explored the link between *P*_DC_ value and secondary structures by comparing the average *P*_DC_ values in the three most populated classes of the CATH classification (hierarchical level *C*: mainly alpha, mainly beta and alpha beta). The ‘mainly alpha’ structure models contained an average 23 ± 13% of *R*_DC_ residues per model, while the ‘mainly beta’ structure models contained fewer *R*_DC_ residues, with on average 13 ± 7% (Fig. 5 ▸). These distributions yielded a significant Kruskal–Wallis test with a *p*-value of 1 × 10^−35^. This test was also associated with an η^2^ value, measuring the effect size of the statistical test, of 0.07 that revealed a moderate effect of the CATH classification on the *P*_DC_ value. The *post hoc* Dunn’s test showed that the average *P*_DC_ values were different in the three main CATH classes (Dunn’s test *p*-value = 2 × 10^−35^ for mainly alpha versus mainly beta, 2 × 10^−10^ for mainly alpha versus alpha beta and 9 × 10^−17^ for mainly beta versus mainly alpha beta). Thus, we concluded that mainly alpha structure models have more questionable conformations on average than other models.

We then explored the relationship between *P*_DC_ values and the year of the model entry, the length, the chain number, the resolution and the crystal system of X-ray structure models (Figs. 5 ▸*b*–5 ▸*f*). Our results showed that the *P*_DC_ values were not related to the year of the structure model entry (*r* = −0.18), the protein length (in terms of amino acids; *r* = 0.35) and its number of chains (*r* = 0.28; Fig. 5 ▸). As illustrated in Figs. 3 ▸ and 5 ▸(*d*), two structure models with different lengths could have the same *P*_DC_ value. For example, the small structure model PDB entry 1emr (R. Robinson, J. Heath, N. Hawkins, B. Samal, E. Jones & C. Betzel, unpublished work; 156 residues) and the large structure model PDB entry 1avo (Knowlton *et al.*, 1997 ▸; 5678 residues) exhibit a *P*_DC_ value close to 50%, meaning that about half of their residues have questionable conformations. Our results also indicated that the *P*_DC_ values were not related to the crystal system of X-ray models (Fig. 5 ▸*b*). Indeed, the seven studied crystal systems exhibited the same average *P*_DC_ value (Kruskal–Wallis test *p*-value = 0.23). As expected, we observed that the *P*_DC_ values were more correlated with the crystallographic data resolution, resulting in a Pearson correlation coefficient of 0.65. It was revealed that the lower the resolution of the X-ray structure model, the greater the *P*_DC_ value. However, two proteins with similar resolutions can exhibit different *P*_DC_ values. Fig. 3 ▸ highlights this observation with the two well resolved models PDB entries 1mhn (Sprangers *et al.*, 2003 ▸) and 2spc (Yan *et al.*, 1993 ▸). Despite their similar resolutions (1.8 Å), their *P*_DC_ values differ considerably: 40% for PDB entry 2spc and 0% for PDB entry 1mhn. The high *P*_DC_ value of PDB entry 2spc indicates many residues with questionable conformations, whereas PDB entry 1mhn had none, showing complete structural conservation between the X-ray and minimized structures. This showed that well resolved structure models could contain lots of residues with questionable conformations. Thus, good resolution does not always mean that there are no questionable conformations. For example, among the 349 structure models with a resolution better than 1.8 Å, eight models had a *P*_DC_ value higher than 20%. In contrast, Fig. 5 ▸(*f*) shows that some structure models with lower resolution (close to 3 Å) exhibit a low *P*_DC_ value. For example, in PDB entry 1bt9 (Phale *et al.*, 1998 ▸; 3 Å resolution, 337 residues), about 13% of the residues are considered to have questionable conformations.

### 3.4. Mapping of residues with questionable conformations in protein structure models

In order to further characterize *R*_DC_ residues, we examined their specific locations within the sequence. Of the 64 571 *R*_DC_ residues detected in the Xray_826_ set, 39% were isolated within sequences, meaning they are flanked by two *R*_CC_ residues. In contrast, the 39 699 remaining *R*_DC_ residues (61%) constituted 14 480 patterns containing between two and 17 *R*_DC_ residues. Most of these patterns (95%) had between two and five *R*_DC_ residues, representing a total of 88% of the *R*_DC_ residues. As expected, when an X-ray model contained a lot of *R*_DC_residues (large *P*_DC_ value), they were grouped in patterns (the Pearson correlation coefficient between the number of patterns and *P*_DC_ is 0.87). Fig. 3 ▸ illustrates *R*_DC_ patterns in three X-ray models with high *P*_DC_ values. In contrast, the number of patterns per structure model showed a moderate correlation with both protein length and the resolution of the crystallo­graphic data (Pearson correlation coefficients of 0.55 and 0.6, respectively). Finally, we showed that the number of patterns was not linked to the X-ray model resolution (the Pearson correlation coefficient is 0.37).

**Figure 6:**
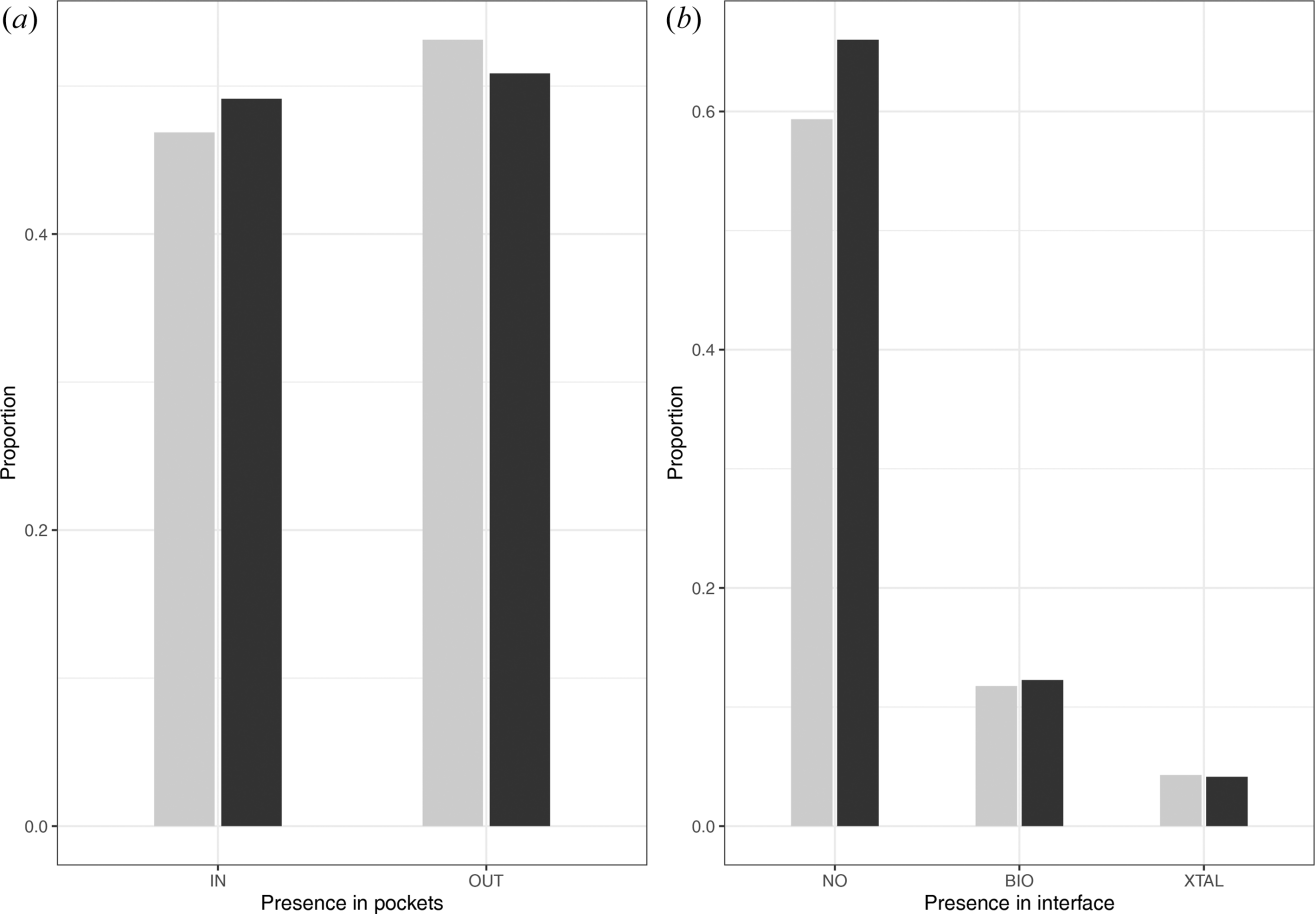
Link between the presence of *R*_DC_ residues and key protein regions. (*a*) Distribution of *R*_CC_ and *R*_DC_ residues located within or outside protein pockets. (*b*) Distribution of *R*_CC_ and *R*_DC_ residues located within or outside protein–protein interfaces. In both panels, *R*_CC_ residues are shown in light gray and *R*_DC_ residues in dark gray.

Next, we investigated the relationship between the presence of *R*_DC_ residues and two key protein regions: protein pockets and protein–protein interfaces. We used the *fpocket* software (Le Guilloux *et al.*, 2009 ▸) to extract pockets from each Xray_826_ structure model. The *PRODIGY-crystal* program (Jiménez-García *et al.*, 2019 ▸) was utilized to extract residues involved in protein–protein interfaces from the 467 oligomeric models of the Xray_826_ set. We then counted the number of *R*_DC_ and *R*_CC_ residues located within pockets and within protein–protein interfaces (Fig. 6 ▸). Almost half of the *R*_DC_ residues (49%) were located in a pocket. Moreover, only a small part of the *R*_DC_ residues (20%) were located within an interface, with 15% in biological interfaces and 5% in crystal interfaces. Fig. 6 ▸ shows that the two types of residues were distributed in a similar way within and outside the pockets or interfaces. These observations were associated with significant Pearson’s χ^2^ tests (*p*-values of 0.001 and 4 × 10^−5^ for pockets and interfaces, respectively) that were accompanied by very low Cramér’s*V* measures close to 0.01. These quantifications indicated a very weak association between the residue types (*R*_DC_ and *R*_CC_) and their location within pockets or interfaces. This revealed that residues with questionable conformations were not preferentially located within pocket or interface regions.

### 3.5. Link between questionable conformations and residue properties

**Figure 7:**
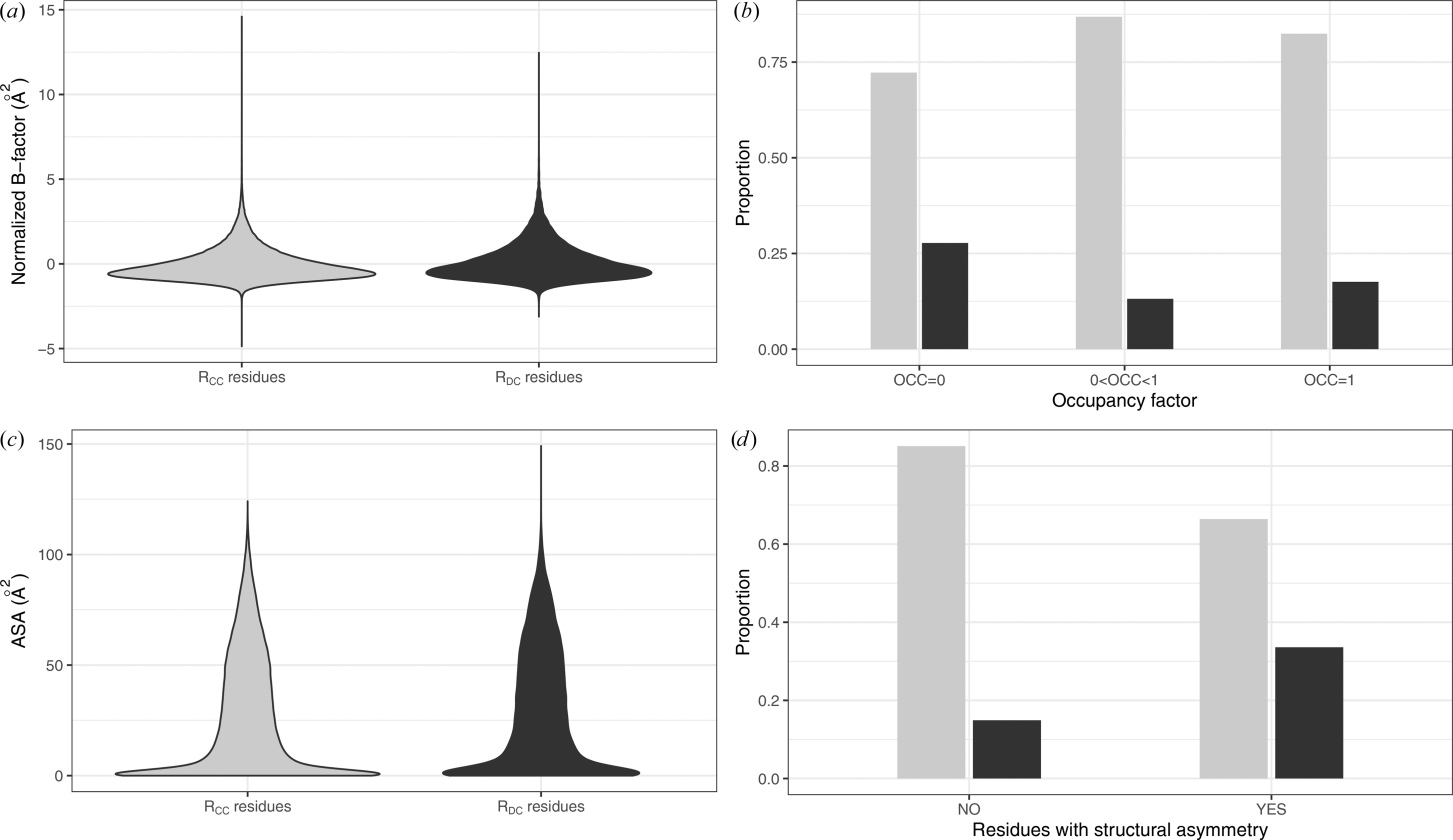
Link between questionable conformations and residue properties. (*a*) Distribution of normalized *B*-factor in the sets of *R*_DC_ and *R*_CC_ residues. (*b*) Distribution of *R*_DC_ and *R*_CC_ residues according to the occupancy-factor value of the residues. (*c*) Distribution of the accessibility surface area (ASA) in the set of *R*_DC_ and *R*_CC_ residues. (*d*) Distribution of *R*_DC_ and *R*_DC_ residues in structurally asymmetric residues and non-asymmetric residues. In all graphs, *R*_CC_ residues are represented in light gray and *R*_DC_ residues are represented in dark gray.

To further investigate residues with questionable backbone conformations, we examined the relationship between *R*_DC_ residues and residue properties (Fig. 7 ▸). We began by focusing on residue flexibility through an analysis of the normalized *B*-factor and occupancy factor of *R*_DC_ and *R*_CC_ residues (Fig. 1 ▸).*R*_DC_ residues had an average normalized *B*-factor of 0.12 ± 0.94 Å^2^, while *R*_CC_ residues exhibited an average normalized *B*-factor of −0.06 ± 1.07 Å^2^ (Fig. 7 ▸*a*). Although the T-test comparing the average *B*-factor values between *R*_DC_ and *R*_CC_ residues resulted in a significant *p*-value (<2 × 10^−12^), the effect size was negligible (a Cohen’s *d* value of −0.18). This indicated a minimal influence of residue type on *B*-factor. These results show that the distributions of normalized *B*-factor values in *R*_DC_ and *R*_CC_ residues were similar. Next, we assessed residue flexibility by analyzing the occupancy factor. Residues were classified into three categories according to their occupancy-factor values: those with an occupancy factor of 0 (representing highly questionable or highly flexible conformations), those with an occupancy factor between 0 and 1 (suggesting at least two alternative conformations) and those with an occupancy factor of 1 (indicating a single conformation). Although the χ^2^ test was significant (Pearson’s χ^2^ test, *p*-value = 3 × 10^−9^), Fig. 7 ▸(*b*) shows that *R*_DC_ and *R*_CC_ residues are globally similarly distributed across the three categories. This is further supported by the very low Cramér’s *V* value of 0.01, indicating no meaningful association between occupancy factor and residue category. Therefore, we concluded that *R*_DC_ residues exhibit similar flexibility to *R*_CC_ residues.

In addition, we explored the relationship between questionable conformations and residue accessibility by comparing the ASA values of *R*_DC_ and *R*_CC_ residues. Fig. 7 ▸(*c*) shows that the ASA distribution in the *R*_DC_ residue set was similar to that in the *R*_CC_ residue set: *R*_DC_ residues had an average ASA value of 29.29 ± 27.05 Å^2^, while *R*_CC_ residues showed an average ASA value of 27.0 ± 26.46 Å^2^. Although the T-test yielded a significant *p*-value (3 × 10^−55^), the Cohen’s *d* value of −0.04 indicated a negligible effect of residue type on ASA values. Consequently, we concluded that *R*_DC_ residues had similar accessibility to *R*_CC_ residues. Our findings did not reveal a significant link between the presence of *R*_DC_ residues and residue flexibility and accessibility. Therefore, questionable conformations do not occur exclusively in highly flexible or highly accessible residues.

We then focused on exploring the relationship between structural asymmetry and questionable conformations. Structural asymmetry occurs in homo-oligomeric proteins, *i.e.* oligomers where the chains share the same amino-acid sequence. It refers to the phenomenon where two chains adopt different conformations (Xiao *et al.*, 1999 ▸; Jin *et al.*, 1999 ▸; Cha *et al.*, 2002 ▸; Renatus *et al.*, 2001 ▸; Triki *et al.*, 2018 ▸; Ollitrault *et al.*, 2018 ▸; Laville *et al.*, 2020 ▸; Badel *et al.*, 2022 ▸). A total of 60 719 structural asymmetric residues were extracted from 309 homo-oligomers of the Xray_826_ set using the HMM-SA protocol developed by Triki *et al.* (2018 ▸) and Ollitrault *et al.* (2018 ▸). We compared the distribution of *R*_DC_ and *R*_CC_ residues among the structurally asymmetric and non-asymmetric residues (Fig. 7 ▸*d*). *R*_DC_ residues were found to be more frequent in asymmetric regions than in symmetric regions. These findings were supported by a significant Pearson’s χ^2^ test (*p*-value ≤ 2 × 10^−16^), but with a weak Cramér’s *V* value of 0.21. This indicated a weak association between residue types (*R*_DC_ and *R*_CC_) and structural asymmetry. In other words, asymmetric residues do not preferentially correspond to residues with questionable conformations.

**Figure 8:**
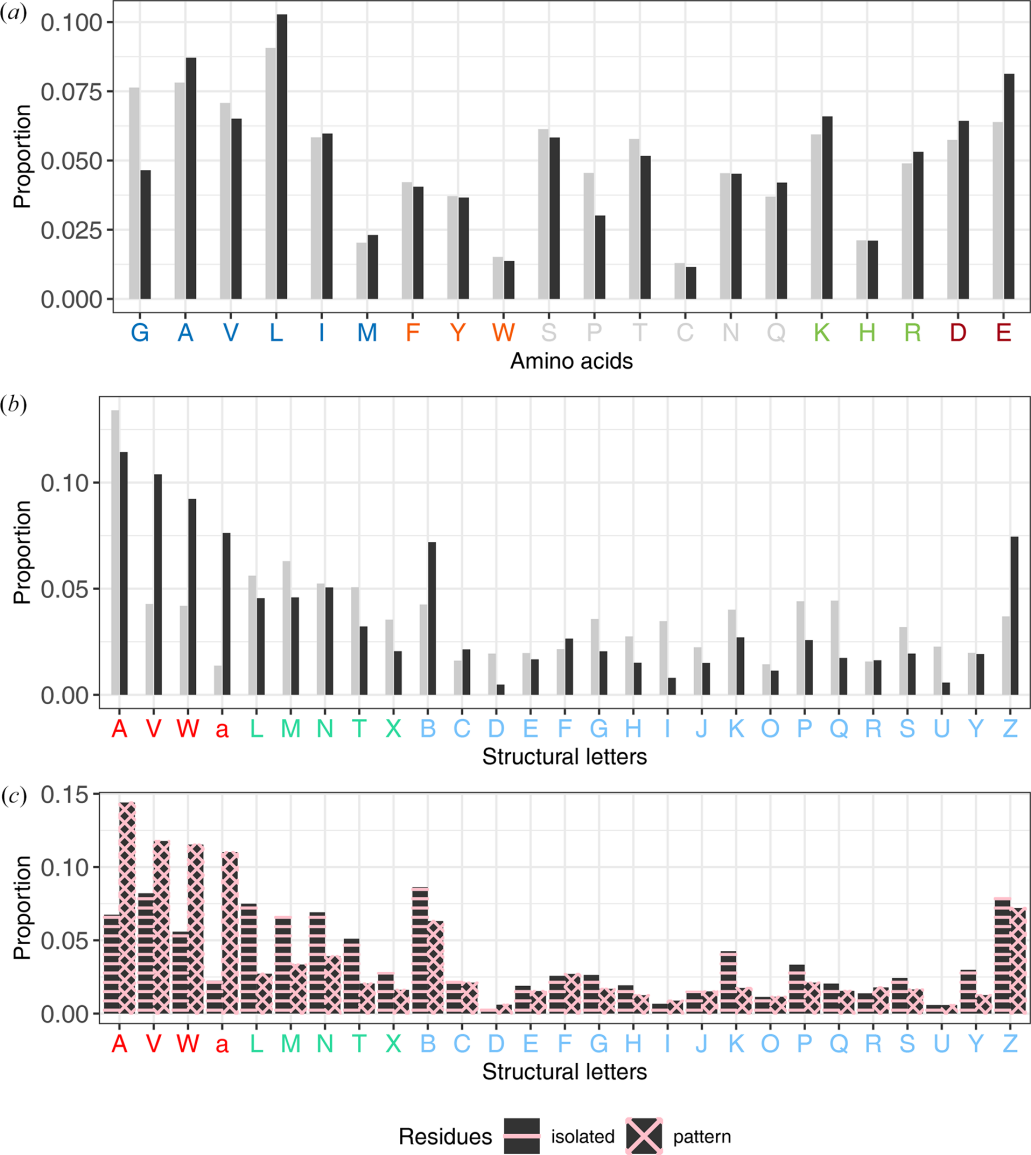
Distribution of the 20 amino acids (*a*) and the 27 structural letters (*b*) within the set of *R*_DC_ and *R*_CC_ residues. (*a*) Amino acids are colored according to their properties: nonpolar aliphatic residues in light blue, aromatic residues in orange, polar uncharged residues in light gray, positively charged residues in green and negatively charged residues in dark red. (*b*) Structural letters are colored according to the secondary structures that they represent: α-helix letters in red, β-strand letters in green and loop letters in blue. Bars are colored according to the residue types: *R*_CC_ residues in light gray and *R*_DC_ residues in dark gray. (*c*) Distribution of the 27 structural letters in isolated *R*_DC_ residues and in *R*_DC_ residues forming patterns.

We then investigated whether questionable conformations occurred more frequently in certain amino acids. To this end, we compared the amino-acid distributions within both the *R*_DC_ and *R*_CC_ residue sets (Fig. 8 ▸*a*). We observed that *R*_DC_ residues were found in all amino acids with a distribution similar to that of *R*_CC_ residues. However, glycine and proline were underrepresented in the *R*_DC_ residue set, while alanine, leucine, lysine and glutamate were overrepresented among residues with questionable conformations (Fig. 8 ▸*a*).

Finally, we explored whether questionable conformations exhibited a preference for specific local conformations. To achieve this, we compared the distribution of the 27 structural letters within both the *R*_DC_ and *R*_CC_ residue sets (Fig. 8 ▸*b*). Our investigation revealed that while all structural letters were present in the *R*_DC_ residue set, their distribution varied significantly compared with the *R*_CC_ residues. Notably, the distribution of α-helix structural letters showed a significant disparity between the two residue sets. Indeed, α-helix conformations were more frequent in the *R*_DC_ residue set, with 39% of *R*_DC_ residues adopting this conformation, compared with only 23% of *R*_CC_ residues (Fig. 8 ▸*b*). Interestingly, the A structural letter is more frequent in *R*_CC_ residues than in *R*_DC_ residues. Moreover, an overrepresentation of the B and Z structural letters was also evident in the *R*_DC_ residue set. Conversely, certain structural letters, including β-strand structural letters (L, M, N, T and X) and the G, H, I, J, K, P, Q and S structural letters, were more frequently observed in *R*_CC_ residues than in *R*_DC_ residues. This outcome confirms that questionable backbone conformations tend to occur with a greater proportion within α-helix regions. To complement our analysis, we examined the distribution of the 27 structural letters in isolated *R*_DC_ residues and those forming patterns (Fig. 8 ▸*c*). We observed that the 27 structural letters were not distributed in the same way in the two types of residues. Indeed, α-helix structural letters were overrepresented in *R*_DC_ residues forming patterns, whereas β-strand structural letters were more commonly found in isolated *R*_DC_ residues. This can be explained by the characteristics of each regular secondary structure. In α-helices, residue *i* forms a hydrogen bond to residue *i* + 4 of the α-helix. Thus, if residue *i* undergoes a conformational change, it can directly impact the conformation of residue *i* + 4 belonging to the α-helix. Furthermore, to detect questionable conformations, we compared the local conformations of residues using HMM-SA. As the structural letters of residues *i* and (*i* + 1) encode the local conformation of the fragments (*i* − 2, *i* − 1, *i* and *i* + 1) and (*i* − 1, *i*, *i* + 1 and *i* + 2), respectively, it is evident that the conformational change induced in residue *i*, altering structural letter *i*, can also modify structural letter (*i* + 1). This explains why we observed an overrepresentation of *R*_DC_ residues forming patterns in α-helices.

### 3.6. Evaluating the effectiveness of our HMM-SA-based protocol for identifying residues with questionable conformations through energy minimization

#### 3.6.1. Comparison with the HMM-SA approach and the r.m.s.d. computation

**Figure 9:**
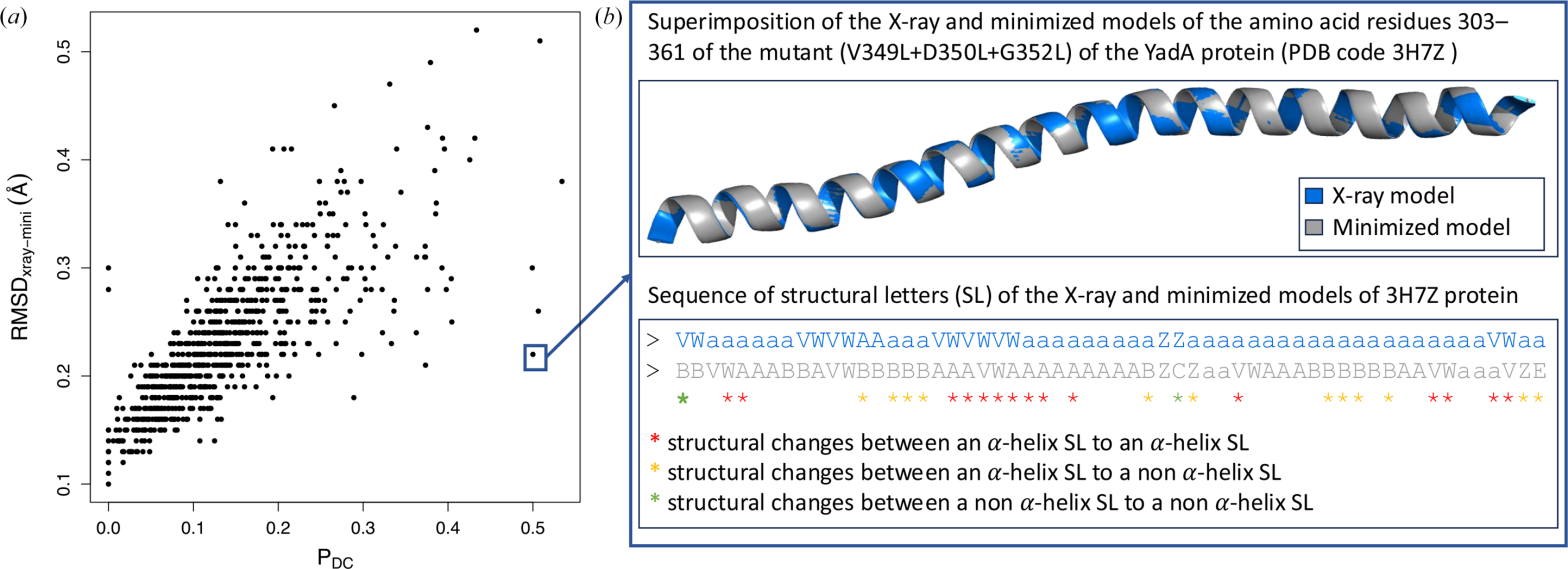
Link between the proportion of residues with questionable conformations and the structural variability between the X-ray and minimized models. (*a*) The relationship between the *P*_DC_ and RMSD_Xray–Mini_ values. (*b*) Illustrations of the proportion of residues with questionable conformations and the structural variability between the X-ray and minimized models of PDB entry 3h7z.

To investigate questionable conformations in X-ray models, some studies have compared X-ray and NMR models of the same protein, or X-ray models resolved under different conditions. These comparisons often rely on computing the r.m.s.d. between the structure models (Betts & Sternberg, 1999 ▸; Andrec *et al.*, 2007 ▸; Mei *et al.*, 2020 ▸; Koehler Leman *et al.*, 2018 ▸; Sikic *et al.*, 2010 ▸). We applied this strategy to the Xray_826_ protein set, measuring the r.m.s.d. between the X-ray and energy-minimized models of each protein (RMSD_Xray–Mini_). The RMSD_Xray–Mini_ values ranged from 0.1 to 0.52 Å, with an average of 0.22 ± 0.06 Å, indicating that energy minimization induces only minor changes in the protein backbone (Supplementary Fig. S3). We then examined the relationship between the proportion of residues with questionable conformations, measured by the *P*_DC_ proportion, and the RMSD_Xray–Mini_ quantifying the structural deviation between X-ray and energy-minimized models (Fig. 9 ▸). We noted a strong positive correlation between these two parameters (Pearson correlation coefficient = 0.78). So, overall, an X-ray model with a high RMSD_Xray–Mini_ value had many residues with questionable conformations. However, some X-ray models had a high *P*_DC_ value despite having a low RMSD_Xray–Mini_. For instance, the ‘mainly alpha’ protein PDB entry 3h7z, corresponding to residues 303–361 of a mutant (V349L+D350L+G352L) of the YadA protein (*Yersinia* adhesin A), illustrates this phenomenon. In this small structure model (58 residues), 50% of residues were detected with questionable conformations (*P*_DC_ = 0.5). However, its X-ray and minimized models were highly similar, with an RMSD_Xray–Mini_ of just 0.22 Å. Except for one residue, all structural changes occurred within α-helix structural letters. For 52% of these residues, the structural changes resulted in an α-helix letter, while the remaining residues adopted a letter close to an α-helix. These results demonstrate that while energy minimization can result in many local conformational changes, the amplitude of these changes is generally very small, leading to low RMSD_Xray–Mini_ values.

#### 3.6.2. Comparison of residues with questionable conformations in PDB/minimized and PDB/PDB-REDO models

**Figure 10:**
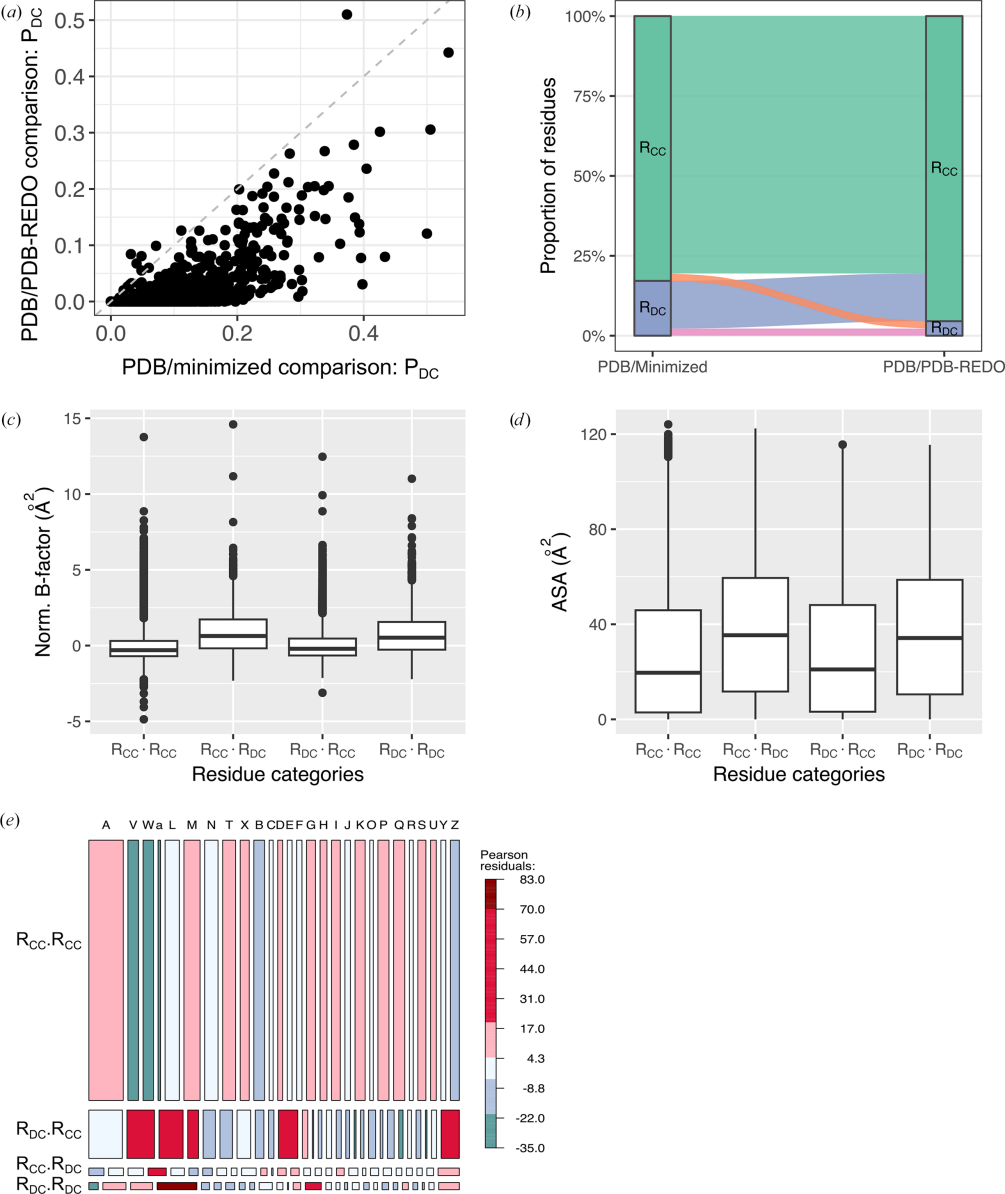
Comparison of residue assignments and proportions of questionable conformations in PDB/minimized and PDB/PDB-REDO models. (*a*) Scatter plot showing the relationship between *P*_DC_ values obtained in the PDB/PDB-REDO comparison and those in the PDB/minimized comparison across the Xray_694_ data set. (*b*) Alluvial plot showing the correspondence between residue classifications in the PDB/minimized (left) and PDB/PDB-REDO (right) comparisons. The rectangular boxes represent residue categories (*R*_CC_ and *R*_DC_), while the colored flows indicate transitions between categories across the two comparisons. The width of each flow is proportional to the fraction of residues undergoing the corresponding transition. (*c*)–(*e*) Exploration of the properties of residues classified based on their assignment (*R*_DC_ or *R*_CC_) in both PDB/minimized and PDB/PDB-REDO comparisons. The *R*_DC_·*R*_DC_ residues are those assigned as *R*_DC_ in both comparisons (PDB/minimized and PDB/PDB-REDO), the *R*_CC_·*R*_CC_ residues are those assigned as *R*_CC_ in both comparisons, the *R*_DC_·*R*_CC_ residues are those assigned as *R*_DC_ in the PDB/minimized comparison and as *R*_CC_ in the PDB/PDB-REDO comparison, and the *R*_CC_·*R*_DC_ residues are those assigned as *R*_CC_ in the PDB/minimized comparison and as *R*_DC_ in the PDB/PDB-REDO comparison. (*c*, *d*) Distributions of the flexibility (*c*), quantified by the normalized *B*-factor, and the accessible surface area (*d*) of the four residue categories. (*e*) Mosaic plot illustrating the distribution of structural letters among the four residue categories. The *x* axis displays the 27 structural letters and the *y* axis displays the four residue categories. The width of the columns is proportional to the number of observations of each structural letter. The vertical length of the bars is proportional to the number of observations in the four residue categories. Pearson residuals from the χ^2^ test: red shades indicate overrepresented combinations (positive residuals), whereas blue shades indicate underrepresented combinations (negative residuals). The intensity of the color is proportional to the magnitude of the residuals.

To further compare our method for identifying residues with questionable conformations with existing approaches, we focused on the PDB-REDO databank (Joosten *et al.*, 2009 ▸, 2014 ▸; Joosten & Vriend, 2007; van Beusekom et al., 2018). The PDB-REDO databank (https://pdb-redo.eu) is an open-access repository that provides refined macromolecular structure models originally obtained from the PDB. The refinement process automatically refines, rebuilds and validates models using the experimental data and established geometric restraints. Among the 826 structure models of the Xray_826_ data set, 694 were available in the PDB-REDO databank. This subset was designated the Xray_694_ set. For these proteins, we had access to three models: the X-ray models (PDB models), the minimized models and the PDB-REDO refined models. We evaluated our approach by detecting *R*_DC_ residues in the Xray_694_ set through two comparisons: (i) X-ray and minimized models (the PDB/minimized comparison) and (ii) X-ray and PDB-REDO models (the PDB/PDB-REDO comparison). Out of the 311 908 Xray_694_ residues, 53 517 residues (17%) were identified as *R*_DC_ in the PDB/minimized comparison, while 14 170 residues (5%) were assigned as *R*_DC_ in the PDB/PDB-REDO comparison. This difference was also apparent at the protein level, as illustrated in Fig. 10 ▸(*a*), which shows the relationship between the proportions of residues with questionable conformations for the PDB/minimized and PDB/PDB-REDO comparisons. Despite a positive linear correlation between the two parameters (*r* = 0.70), 97% of the X-ray models displayed lower *P*_DC_ values in the PDB/PDB-REDO comparison than in the PDB/minimized comparison. These results indicated that the structure models from the PDB-REDO databank were structurally closer to the original X-ray structures from the PDB than to those subjected to energy minimization. Thus, as expected, energy minimization induced more conformational changes in the backbone than the refinement process of the *PDB-REDO* protocol.

To further investigate, we compared the assignment (*R*_DC_or *R*_CC_) of the 311 908 residues across the two comparisons (PDB/minimized and PDB/PDB-REDO), as shown in Fig. 10 ▸(*b*). Among the 14 170 residues classified as *R*_DC_ in the PDB/PDB-REDO comparison, 49% retained the same classification in the PDB/minimized comparison. As expected, this proportion decreased when considering the reverse comparison: only 12% of residues labeled as *R*_DC_ in the PDB/minimized comparison were also assigned as *R*_DC_ in the PDB/PDB-REDO comparison. To explore those residues assigned differently across the two comparisons in more detail, we compared the properties (flexibility, solvent accessibility and local conformations) of the four residue categories identified in the two comparisons: (i) *R*_DC_·*R*_DC_, residues assigned as *R*_DC_ in both comparisons, (ii) *R*_CC_·*R*_CC_, residues assigned as *R*_CC_ in both comparisons, (iii) *R*_DC_·*R*_CC_, residues assigned as *R*_DC_ in the PDB/minimized comparison and as *R*_CC_ in the PDB/PDB-REDO comparison, and (iv) *R*_CC_·*R*_DC_, residues assigned as *R*_CC_ in the PDB/minimized comparison and as *R*_DC_ in the PDB/PDB-REDO comparison (Figs. 10 ▸*c*–10 ▸*e*). We first examined whether the four residue categories differed in their flexibility (normalized *B*-factors) and solvent accessibility (ASA). The Kruskal–Wallis test yielded significant results (*p*-value ≤ 2.2 × 10^−16^). However, the four residue categories showed a similar distribution (Fig. 10 ▸*c*), and the very small effect sizes (η^2^ = 0.02 and 0.006) confirmed only a weak relationship between the residue category and both flexibility and solvent accessibility. These results suggested that residues with different assignments between the two comparisons were not among the most flexible or solvent-accessible residues.

To further our analysis, we investigated whether residues assigned differently in the two comparisons were preferentially associated with specific local conformations. To this end, we analyzed the distribution of the 27 structural letters across the four categories of residues (*R*_CC_·*R*_CC_, *R*_DC_·*R*_DC_, *R*_CC_·*R*_DC_ and *R*_DC_·*R*_CC_; Fig. 10 ▸*e*). Our results revealed significant differences in the distribution of the 27 structural letters among the four residue categories (Pearson’s χ^2^ test, *p*-value ≤ 2.2 × 10^−16^). The effect size was small to moderate (Cramér’s *V* = 0.17), indicating only a weak-to-moderate association between the 27 structural letters and the four residue categories. We paid particular attention to the structural letters associated with α-helices. We observed that letters a, V and W were overrepresented in *R*_DC_·*R*_DC_ and *R*_DC_·*R*_CC_ residues. This indicated that questionable conformations were frequently found in these residues during the PDB/minimized comparison but were not always confirmed by the PDB/PDB-REDO comparison. Residues adopting letter a showed a different behavior in *R*_DC_·*R*_DC_ and *R*_CC_·*R*_DC_ residues (Fig. 10 ▸*e*). In fact, letter a was even more strongly overrepresented than V and W in *R*_DC_·*R*_DC_, and it was also overrepresented in *R*_CC_·*R*_DC_. Specifically, 1100 residues adopting the a conformation were identified as *R*_DC_ in the PDB/minimized comparison, whereas only 135 would be expected under the assumption of independence between structural letters and residue categories. Among all structural letters, a also exhibited the highest agreement rate for *R*_DC_ residues across both comparisons, with 35% of a-assigned residues consistently classified as *R*_DC_. Furthermore, of the 1626 residues assigned the a conformation and categorized as *R*_DC_ in the PDB/PDB-REDO comparison, more than 68% were reassigned as *R*_DC_ in the PDB/minimized comparison. This suggested that residues adopting the a conformation, corresponding to a highly regular α-helical geometry, were more frequently found at positions that appear prone to structural adjustments following refinement or energy-minimization steps. The structural letter A was notably underrepresented in *R*_DC_·*R*_DC_ residues and overrepresented in residues classified as *R*_CC_·*R*_CC_, with more than 83% of all A-assigned residues falling into this category. This suggested that residues adopting the A conformation tended to retain their classification across both comparisons, and might correspond to conformations that were stable under both re-refinement and energy-minimization procedures. In other words, these positions appeared to be less susceptible to model-dependent variability, possibly reflecting structurally rigid or well defined regions.

While the letters a and A both correspond to α-helical conformations, they displayed different distributions in the four residue categories. To explore whether these differences were related to distinct dynamic properties, we compared the normalized *B*-factors of residues assigned to a and A across the full Xray_694_ data set. Residues with the a conformation exhibited higher average *B*-factors (0.006 ± 0.99 Å^2^) than those assigned to A (−0.26 ± 0.74 Å^2^). This difference, although significant (T-test *p*-value = 2.5 × 10^−86^), was associated with only a small effect size (Cohen’s *d* = 0.34). For example, 45% of a-assigned residues classified as *R*_DC_·*R*_DC_ were rigid, while 25% of A-assigned residues in the *R*_CC_·*R*_CC_ category were flexible. These observations suggested that the presence of the structural letter a might indicate ambiguous or flexible regions that were capable of undergoing conformational changes during energy minimization or re-refinement. In contrast, the letter A appeared to mark regions whose geometry was less sensitive to the modeling protocol, and thus were more structurally stable.

### 3.7. Case study: exploration of questionable conformations in PR2

#### 3.7.1. Identification of residues with questionable conformations in the structure model of the ligand-free form of PR2

The HIV-2 protease is an important target to treat HIV-2 infection. It is a small homodimer of 99 residues per chain which is involved in the maturation of the virus (Menéndez-Arias & Álvarez, 2014 ▸). This target adopts a semi-open conformation in the absence of the ligand. Upon ligand binding, the binding site closes via the flap regions, resulting in the closed form of PR2. In this form, the ligand adopts its proper orientation in the catalytic site (Gustchina & Weber, 1991 ▸; Kar & Knecht, 2012 ▸; Chen *et al.*, 2014 ▸). In the PDB, 19 X-ray models are available: 18 are complexed with an inhibitor and one is in a ligand-free form without the ligand (PDB entry 1hsi; Chen *et al.*, 1994 ▸). In this section, we explored questionable conformations occurring in the ligand-free form of PR2 by applying our HMM-SA-based protocol to PDB entry 1hsi. Our protocol highlighted 32 *R*_DC_ residues: 18 and 14 *R*_DC_ residues in chains *A* and *B*, respectively (Fig. 1 ▸). Thus, 16% of the residues in the X-ray model of the ligand-free PR2 protein exhibited local questionable conformations. However, for 70% of these *R*_DC_ positions the structural letters extracted from both the X-ray and minimized models corresponded to the same secondary structure. This indicates that these structural changes induced by minimization were of weak magnitude (Fig. 1 ▸). Furthermore, 56% of these *R*_DC_ positions were organized into seven patterns spanning two to four *R*_DC_ residues. Additionally, Fig. 1 ▸ shows that certain *R*_DC_ residues were found in flexible regions, such as the flap region, while others were located in rigid regions, such as the α-helix region. More precisely, 59% of *R*_DC_ residues were found in flexible regions, defined according to their *B*-factor values. Interestingly, the *R*_DC_ positions did not coincide across the two chains (Fig. 1 ▸), despite these chains sharing identical amino-acid sequences. This reinforces the fact that PR2 is a protein in which the two chains do not have the same properties. Specifically, we identified 34 structurally asymmetric positions, *i.e.* having different local conformation in both chains, using the HMM-SA approach (Ollitrault *et al.*, 2018 ▸). Of these residues, 15 were *R*_DC_residues in at least one of the two chains.

#### 3.7.2. Link with questionable conformations and structural outliers

**Figure 11:**
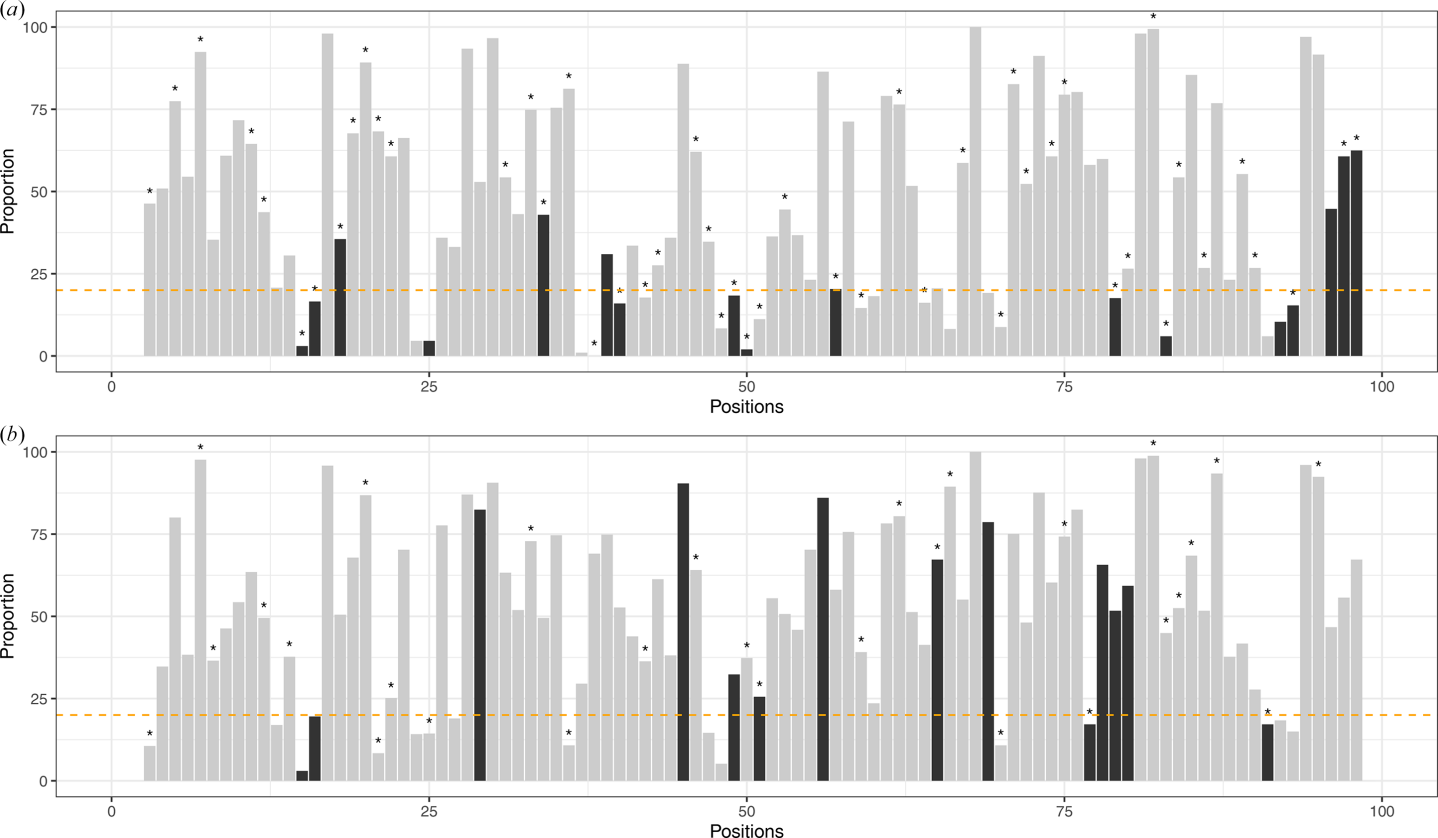
Exploring the structural landscape of the PR2 structure models using molecular-dynamics simulations. The numbers of MD models containing the same structural letter as PDB entry 1hsi in chains *A* (*a*) and *B* (*b*). Positions with questionable conformations (*R*_DC_ positions) in the X-ray model are highlighted in dark gray. Stars identify positions having structural outliers according to the wwPDB X-ray Structure Validation Report for PDB entry 1hsi.

To validate whether the *R*_DC_ positions observed in PDB entry 1hsi correspond to structural outliers, we analyzed the wwPDB X-ray Structure Validation Report of PDB entry 1hsi (https://files.rcsb.org/pub/pdb/validation_reports/hs/1hsi/1hsi_full_validation.pdf.gz). This report identified 81 residues containing at least one outlier in geometric quality criteria, named structural outliers. 18 of these structural outliers were detected as *R*_DC_ residues (56% of *R*_DC_ residues; Fig. 11 ▸). These results reinforce the hypothesis that these *R*_DC_ residues correspond to structural outliers with questionable conformations. In contrast, 14 *R*_DC_ residues were not detected as structural outliers. Most of these *R*_DC_ residues correspond to the residues before or after *R*_DC_ outlier residues. We also identified 63 outlier residues that were not classified as *R*_DC_ residues.

To continue the analysis, we performed a molecular-dynamics simulation (500 ns) of PDB entry 1hsi to sample a large part of its conformational space. A set of 501 frames was extracted from this simulation. These structure models were referred to as MD models. Using HMM-SA (Camproux *et al.*, 2004 ▸), we examined the local conformations, represented by the structural letters, at each position in the 501 MD models. To determine whether the local conformations extracted from PDB entry 1hsi were frequently sampled during the simulation, we counted, for each position, the number of MD models that exhibited the same structural letter as that extracted from PDB entry 1hsi (Fig. 11 ▸). Based on this criterion, we categorized the *R*_DC_ residues from PDB entry 1hsi into two distinct groups. Firstly, 15 *R*_DC_ residues (47%) had a structural letter in PDB entry 1hsi that was observed in less than 20% of the MD models, revealing that these local conformations were uncommon and rather rare. This outcome confirms that these *R*_DC_ positions correspond to conformations that are unlikely to represent the predominant state, which supports their characterization as residues with questionable conformations. In contrast, other *R*_DC_ positions exhibited structural letters in PDB entry 1hsi that were also observed in at least 20% of the MD models. These local conformations were commonly observed throughout the trajectory, highlighting their high likelihood, particularly for the three positions which displayed identical structural letters to PDB entry 1hsi in over 80% of the MD models. These findings suggest that these local backbone conformations correspond to a well supported conformation, despite initially being identified as questionable conformations by our protocol. One hypothesis that could explain this result is the fact that these positions could adopt multiple biological conformations. For example, the *R*_DC_ residue at position 56 of chain *B* exhibited structural letters X and N in the X-ray and minimized models, respectively. During the simulation, 86% of MD models had the X structural letter at this position, 12% had N and less than 2% adopted other letters (J, R or T). We thus supposed that the two conformations encoded by the X and N structural letters corresponded to two possible well supported conformations. Furthermore, Fig. 11 ▸ identified 24 *R*_CC_ positions where the structural letters observed in the X-ray model were present in less than 20% of MD models. This result suggested that these local conformations of PDB entry 1hsi were uncommon and rare, indicative of a questionable conformation. Consequently, the energy-minimization step was not sufficient to reveal these questionable conformations.

#### 3.7.3. Impact of questionable backbone conformations on the PR2 fold

**Figure 12:**
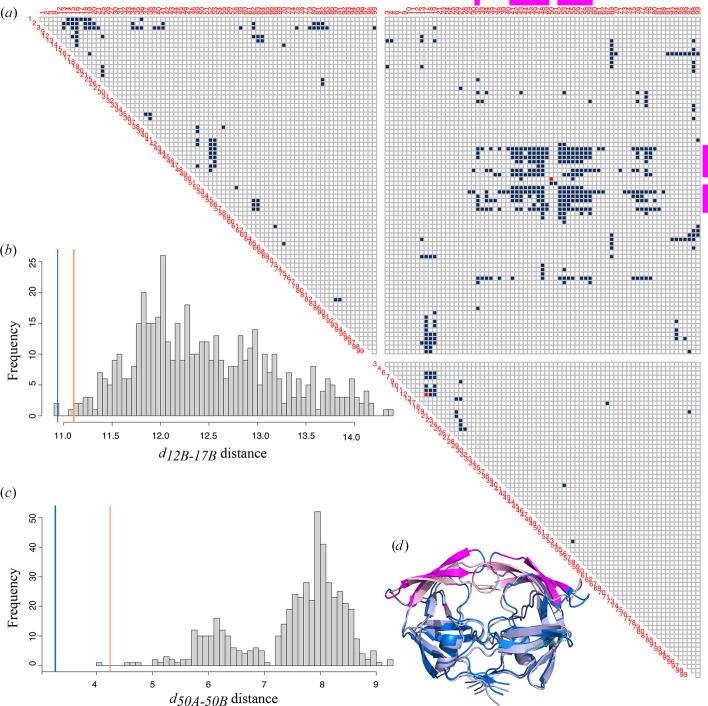
Comparison between the structural fold of X-ray and MD models of the free form of PR2 based on the intra-distances. (*a*) Matrix highlighting residue pairs (in blue) for which the intra-distance values in the X-ray model (PDB entry 1hsi) were excluded from the interval comprising 99% of values determined in the MD model set, denoted as IC_MD_. Only residues involved in at least one pair having a distance in PDB entry 1hsi excluded from IC_MD_ were represented in the matrix. Magenta highlights regions where PDB entry 1hsi exhibited an outlier intra-distance compared with the MD models, *i.e.* having a lot of residues involved in pairs with a distance in PDB entry 1hsi excluded from IC_MD_. (*b*, *c*) Distribution of the *d*_12*B*–17*B*_ and *d*_50*A*–50*B*_ distances in the MD model set. Blue and orange lines correspond to the distance values computed on the X-ray and minimized models of PR2, respectively. (*d*) Illustration of distances *d*_12*B*–17*B*_ and *d*_50*A*–50*B*_ in PDB entry 1hsi (marine blue) and the MD model extracted at 150 ns (light blue). Regions colored in magenta correspond to regions with a lot of outlier distances in PDB entry 1hsi.

To assess the impact of these questionable conformations on the global PR2 conformation, we calculated the 19 503 distances between each C^α^ atom in all MD models and for PDB entry 1hsi. For each distance, we determined the confidence interval IC_MD_, corresponding to the range containing 99% of the values computed in the MD model set. We then compared the distances measured in PDB entry 1hsi with these IC_MD_ intervals. Distances from the X-ray model that lay outside these intervals were considered outliers. In total, 508 distances (3%) were identified as outliers (Fig. 12 ▸*a*). This indicated that these distances in the X-ray model deviated from the range observed during molecular dynamics, thereby characterizing them as outliers. These results suggested that the residues involved in these distances might adopt atypical or misfolded conformations in the X-ray model. Figs. 12 ▸(*b*) and 12 ▸(*c*) illustrate the distribution of two of these distances, the distances *d*_12*B*–17*B*_ and *d*_50*A*–50*B*_, which were the distances computed between the C^α^ atoms of residues 12 and 17 of the *B* chain and between the C^α^ atoms of residues 50 in both chains, respectively. In the MD model set, the *d*_12*B*–17*B*_ distance varied from 10.91 to 14.36 Å, with an average value of 12.47 ± 0.73 Å and an IC_MD_ interval equal to [11.11; 14.17 Å]. We noticed that this IC_MD_ interval did not contain the *d*_12*B*–17*B*_ distance value calculated for PDB entry 1hsi (*d*_12*B*–17*B*_ = 10.94 Å). We observed similar results for the *d*_50*A*–50*B*_ distance. Indeed, its IC_MD_ interval is [4.67; 8.98 Å], while PDB entry 1hsi had a *d*_50*A*–50*B*_ distance of 3.27 Å. More precisely, we noted that in all 501 MD frames the *d*_50*A*–50*B*_ distance was larger than in the X-ray model. This highlighted that no MD model adopted a conformation that reflected the *d*_50*A*–50*B*_ distance computed in the X-ray model. The 508 outlier distances involved residues distributed throughout the protein (Fig. 12 ▸*a*). However, there was an overrepresentation of residues between the two chains, especially in regions 42–57 for chain *A* and 41–59 for chain *B*. Visualizing these residues on the PR2 X-ray model revealed that they were located in the flap regions, which close over the binding site (Fig. 12 ▸*d*). These results suggested that the flap position in the PR2 X-ray model was questionable.

## 4. Discussions and conclusion

In this study, we explored and quantified questionable backbone conformations in X-ray structure models. Traditionally, the impact of the crystallographic process on protein structures is studied by comparing X-ray models resolved under different conditions or NMR and X-ray models of the same target (Betts & Sternberg, 1999 ▸; Jacobson *et al.*, 2002 ▸; Eyal *et al.*, 2005 ▸; Garbuzynskiy *et al.*, 2005 ▸; Andrec *et al.*, 2007 ▸; Sikic *et al.*, 2010 ▸; Koehler Leman *et al.*, 2018 ▸; Mei *et al.*, 2020 ▸; Grigas *et al.*, 2022 ▸). For example, the study of Garbuzynskiy and coworkers compared NMR and X-ray models of 78 proteins (Garbuzynskiy *et al.*, 2005 ▸). Similarly, the studies of Andrec and coworkers and Sikic and coworkers examined the effect of crystal packing by comparing 148 and 109 pairs of NMR and X-ray models, respectively (Andrec *et al.*, 2007 ▸; Sikic *et al.*, 2010 ▸). More recently, in their study, Mei and coworkers compared the cores of X-ray and NMR models for 21 proteins, revealing that NMR models are more tightly packed than the cores of X-ray models (Mei *et al.*, 2020 ▸). To increase the size of the data set, Grigas and coworkers used pairs of X-ray and NMR models, defined as such when the two models shared more than 90% sequence similarity (Grigas *et al.*, 2022 ▸). Koehler Leman and coworkers explored the impact of using different experimental methods to resolve the structures of 14 membrane proteins (Koehler Leman *et al.*, 2018 ▸). Other studies focused on the comparison of structure models of the same protein in different crystal environments. In particular, Eyal and coworkers built three data sets to investigate the effect of the crystal environment on a protein structure model (Eyal *et al.*, 2005 ▸). The first data set contained 404 pairs of structure models of the same protein obtained from different crystals but sharing identical crystal forms. It was used to quantify inaccuracies in structure determination. The two other sets corresponded to model pairs of the same proteins where the environment of the two molecules was different. One of these data sets contained 107 pairs extracted from structure models with two molecules found in the asymmetric unit of a single-crystal structure model. The last set was composed of 148 paired models whose members came from different crystals exhibiting distinct crystal forms. These two latter data sets measured the influence of crystal packing versus crystallization conditions, such as pH, temperature and ligand occupancy, on protein structure models. By comparing the global conformation and side-chain conformations in the three data sets, they showed that the crystal environment induces deformations in the backbone and side chains, potentially leading to hinge-like motions. They also observed that the crystal environment impacts the positions of water molecules, but not those of the ligands. These structural changes are also induced by the use of different refinement methods. In several studies, authors built a set containing X-ray models independently crystallized by different groups as a control set (Martin *et al.*, 2008 ▸; Mei *et al.*, 2020 ▸). For example, to distinguish structural deformations induced by partner binding and experimental errors, Martin and coworkers used a set of 14 protein pairs independently crystallized by different groups (Martin *et al.*, 2008 ▸). In their study, Mei and coworkers constructed an X-ray duplicate (same crystal forms and space groups) data set of 39 proteins from the PDB to evaluate structural variations corresponding to errors in X-ray structure models (Mei *et al.*, 2020 ▸). Unfortunately, in the PDB there are few protein models resolved by both NMR and X-ray crystallography and it is difficult to compare a multitude of NMR models with a single X-ray model. Apart from a few studies, most analyses focused on small data sets. To address this issue, we developed a protocol to identify questionable conformations by comparing local conformations in X-ray models before and after energy minimization. The underlying assumption is that energy minimization optimizes the atomic geometry of a structure model by reducing the potential energy, resulting in a physically plausible and stable conformation close to a minimal energy state. Using this protocol, we were able to study questionable conformations in a large data set composed of 826 X-ray models.

To extract backbone local conformations in X-ray and minimized models, we used the HMM-SA structural alphabet (Camproux *et al.*, 2004 ▸). We have previously demonstrated that HMM-SA is effective for investigating local deformations induced by protein–protein interactions (Martin *et al.*, 2008 ▸), by ligand binding (Regad *et al.*, 2017 ▸; Triki *et al.*, 2018 ▸, 2019 ▸; Laville *et al.*, 2020 ▸; Baillif *et al.*, 2025 ▸; Camproux *et al.*, 2025 ▸), by intrinsic flexibility (Ollitrault *et al.*, 2018 ▸) or by mutations (Regad *et al.*, 2017 ▸; Triki *et al.*, 2020 ▸; Camproux *et al.*, 2025 ▸). In our protocol, HMM-SA was used to simplify X-ray and minimized models into 1D sequences of structural letters, where each structural letter corresponds to the fold of a fragment of four residues. Thus, the comparison of X-ray and minimized models was reduced to comparing structural-letter sequences. To select only structural changes of meaningful amplitude, only residues that exhibited different structural letters between the two models and showed a structural deviation greater than 0.1 Å were retained. Consequently, residues with questionable conformations were defined as those having different structural letters in the X-ray and minimized models and RMSD_frag_ > 0.1 Å. This approach allowed the rapid identification of residues with questionable backbone conformations. Our protocol, applied to the 826 models of the Xray_826_ set, revealed that, on average, an X-ray model contains 18% of residues with questionable backbone conformations. We noted that 39% of residues with questionable backbone conformations were isolated in the amino-acid sequences, while 61% formed patterns of 2–17 residues. This quantification is in agreement with previous studies. A comparison of X-ray models of 14 proteins solved by different groups showed that changes in the crystallographic environment accounted for structural differences in 28% of residues per model (Martin *et al.*, 2008 ▸). Similarly, structural motions induced by ligand binding were identified by comparing X-ray models of identical proteins in ligand-bound and unbound forms (Amemiya *et al.*, 2011 ▸, 2012 ▸). These motions predominantly occurred near the co-crystallized ligands, and up to 30% of observed conformational changes (both domain and local motions) were attributed to changes in the crystal environment rather than ligand binding (Amemiya *et al.*, 2011 ▸, 2012 ▸). The analysis of the location of questionable backbone conformations in the Xray_826_ model set revealed that questionable conformations were more frequent in α-helix regions, while they were underrepresented in loop regions. Residues with questionable conformations were enriched in alanine, leucine and glutamate. This enrichment reflects the large proportion of questionable conformations occurring in α-helices. Indeed, it is known that alanine, leucine and glutamate have a high propensity to form α-helices (Pace & Scholtz, 1998 ▸). Our results, based on a large data set, are consistent with our previous findings obtained by comparing X-ray models of 14 proteins determined by different groups (Martin *et al.*, 2008 ▸). In that study, we showed that experimental errors and protein flexibility mainly affect helices, less frequently strands and only occasionally loops. However, these findings do not agree with those obtained by Sikic *et al.* (2010 ▸) and Eyal *et al.* (2005 ▸), who compared, using global r.m.s.d., the structures of 109 NMR/X-ray model pairs and X-ray models of the same protein solved in different crystal environments, respectively (Sikic *et al.*, 2010 ▸; Eyal *et al.*, 2005 ▸). They demonstrated that loop regions are more variable, while β-strands exhibit more conserved conformations and α-helices display intermediate variability. This inconsistency between these studies and our results can be attributed to methodological differences in how structural changes were analyzed and quantified.

To further assess our method for analyzing residues with questionable conformations in X-ray models, we compared the *P*_DC_ and RMSD_Xray–Mini_ parameters, both of which are derived from the comparison of X-ray and minimized models. Our analysis revealed that the RMSD_Xray–Mini_ values for the Xray_826_ data set were generally low, indicating that energy minimization induced only minor adjustments to the protein backbone. In addition, we noted that proteins exhibiting higher structural deviations between X-ray and minimized models often contain more residues with questionable conformations. However, a high *P*_DC_ does not necessarily correlate with a high RMSD_Xray–Mini_, particularly in ‘main alpha’ protein. This discrepancy can be explained by the distinct nature of these two metrics: *P*_DC_ quantifies the number of local conformational changes, while RMSD_Xray–Mini_ measures the magnitude of overall structural deviations. However, our HMM-SA approach achieved precise localization and quantification of questionable conformations in X-ray models without requiring optimal superposition of the models, unlike classical approaches such as r.m.s.d. or *TM-align* score calculation (Betts & Sternberg, 1999 ▸; Andrec *et al.*, 2007 ▸; Mei *et al.*, 2020 ▸; Koehler Leman *et al.*, 2018 ▸; Sikic *et al.*, 2010 ▸). However, one limitation of our approach is its focus on backbone conformations, excluding questionable conformations related to side-chain positions. Future adaptations of HMM-SA could integrate side-chain flexibility to provide a more comprehensive picture of structurally questionable conformations.

To validate our approach, we compared two strategies to identify questionable residues: (i) PDB X-ray versus minimized models and (ii) PDB X-ray versus PDB-REDO models. PDB-REDO models correspond to re-refined versions of PDB entries in which automated procedures improve stereochemistry and the fit to experimental data while preserving the crystallographic environment. As expected, the latter comparison identified fewer residues with questionable conformations than the PDB/minimized comparison. Notably, 50% of the questionable conformations detected in the PDB/PDB-REDO comparison were also identified in the PDB/minimized comparison. This result reflects the fact that *PDB-REDO* applies targeted refinements and geometric adjustments, while respecting the original experimental data. It corrects local errors without introducing major deviations. In addition, *PDB-REDO* does not modify the crystal-packing environment, and thus conformational biases induced by packing contacts remain unchanged. Energy minimization, in contrast, can produce global changes since crystallographic restraints are not enforced. This comparison confirms the validity of our approach, showing that both strategies converge, while the PDB-REDO models, being closer to the experimental data, yield fewer questionable residues than the minimized models. At the structural-letter level, residues encoded by the letter A were enriched among well defined conformations with higher confidence, while residues encoded by the letter a were enriched among questionable conformations in both comparisons. Residues with the A conformation generally show lower flexibility than those with the a conformation, but flexibility alone does not explain this contrast. Structural letter a may in part reflect artifacts introduced by refinement methods used in the past, such as simulated annealing in *X-PLOR* or *CNS*, which are rarely employed today. Supporting this idea, we found that the letter a is overrepresented in structure models refined with *X-PLOR* in the PDB (data not shown). However, the association between structural letters and refinement software is weak, and letter a cannot be considered a pure artifact. It likely reflects both a historical methodological bias and the presence of rare conformations. It would be interesting to conduct a deeper study to investigate this link.

We then investigated the relationship between *P*_DC_ values and X-ray model properties. Our analysis indicated that the proportion of questionable conformations does not depend on either the year of structure model deposition or the protein length. This result is in agreement with the study of Andrec and coworkers, where the authors compared the global fold of 148 NMR/X-ray model pairs (Andrec *et al.*, 2007 ▸). We also found that there is a moderate correlation between the resolution of the crystallographic data and the proportion of questionable conformations. A similar result was reported by Mei and coworkers, who revealed that the magnitude of the deformation between X-ray and NMR model pairs is not strongly correlated with the resolution of the X-ray models (Mei *et al.*, 2020 ▸). Thus, even well resolved models can contain a lot of questionable conformations, and structure models with a resolution lower than 3 Å are not always those with the most questionable conformations, as mentioned in the study by Davis *et al.* (2008 ▸). In their study, the authors explained that even in well resolved models some regions of the electron density may be poorly defined or open to different interpretations. Such differences can lead to biases, ambiguities and errors in atom positions and residue conformations (Davis *et al.*, 2008 ▸). An analysis of the location of questionable conformations revealed no link between the presence of a questionable conformation of a residue and its flexibility or accessibility. In addition, we showed that questionable conformations did not preferentially occur in pockets or protein–protein interfaces. In other words, questionable backbone conformations were not more likely to occur in regions prone to deformation.

In the first part of our study, we explored questionable conformations in a large set of structure models. In the next part of the study, we investigated in detail the link between the presence of questionable conformations and outliers and rare conformations. To this end, we located questionable conformations in HIV-2 protease (PR2), an enzyme involved in virion maturation and therefore important for the treatment of HIV-2 infection. The application of our protocol to the X-ray model of PR2 in the ligand-free form (PDB entry 1hsi) enabled us to locate 32 residues with questionable conformations. We showed that 65% of these residues with questionable conformations matched the structural outliers reported in the wwPDB X-ray Structure Validation Report or adopted conformations that were rarely observed during molecular-dynamics simulations. These results strengthen the evidence that these residues correspond to questionable conformations. Interestingly, in PDB entry 1hsi we also identified questionable conformations which corresponded to a conformation that was frequently sampled during molecular-dynamics simulations. For these residues, the questionable nature of their conformation therefore appears less evident. This could be explained by the fact that these residues are present in two stable conformations corresponding to the two different conformations in the X-ray and minimized models. Conversely, we identified X-ray residues that our protocol characterized as having a well defined and reliable conformation, yet this conformation was only rarely sampled during molecular-dynamics simulations. This suggests that our protocol does not allow us to highlight residues whose conformations appear questionable when assessed through molecular-dynamics approaches. It would be interesting to extend the minimization step and analyze these residues in more detail. Finally, we studied the impact of these local questionable conformations on the global conformation of PR2, based on the intra-atomic distances calculated in the X-ray and MD models. This study showed that 3% of these distances computed on PDB entry 1hsi had singular values that were rarely observed during simulation. These distances mainly involved residues located in the flaps of both chains. Consequently, in PDB entry 1hsi these regions seem to adopt an atypical relative position, possibly reflecting the influence of the crystallographic environment. This raises the question of the biological relevance of the flap shape in PDB entry 1hsi, theonly model of the ligand-free form of PR2 available in the PDB.

The analysis and comparison of X-ray models are often used to characterize protein function, to study the impact of mutations and to search for new inhibitors. Our study showed that questionable conformations can be frequent events, particularly in models with many α-helices. We have shown that these questionable conformations can lead to outlier conformations and thus have a negative impact on structural analyses and distort results. Our analyses highlight the importance of considering potential questionable conformations in X-ray models and emphasize the need for a careful and thorough assessment of their quality before use.

## Data Availability

Data supporting the results of our manuscript are available at https://owncloud.rpbs.univ-paris-diderot.fr/owncloud/index.php/s/1NqUDvSPdPK0zcZ
